# Comparative Mitogenomics of Plant Bugs (Hemiptera: Miridae): Identifying the AGG Codon Reassignments between Serine and Lysine

**DOI:** 10.1371/journal.pone.0101375

**Published:** 2014-07-02

**Authors:** Ying Wang, Hu Li, Pei Wang, Fan Song, Wanzhi Cai

**Affiliations:** 1 Department of Entomology, China Agricultural University, Beijing, China; 2 Department of Ornamental Horticulture, China Agricultural University, Beijing, China; 3 Key Laboratory of Molluscan Quarantine and Identification of AQSIQ, Fujian Entry-Exit Inspection & Quarantine Bureau, Fuzhou, Fujian, China; University of Iceland, Iceland

## Abstract

Insect mitochondrial genomes are very important to understand the molecular evolution as well as for phylogenetic and phylogeographic studies of the insects. The Miridae are the largest family of Heteroptera encompassing more than 11,000 described species and of great economic importance. For better understanding the diversity and the evolution of plant bugs, we sequence five new mitochondrial genomes and present the first comparative analysis of nine mitochondrial genomes of mirids available to date. Our result showed that gene content, gene arrangement, base composition and sequences of mitochondrial transcription termination factor were conserved in plant bugs. Intra-genus species shared more conserved genomic characteristics, such as nucleotide and amino acid composition of protein-coding genes, secondary structure and anticodon mutations of tRNAs, and non-coding sequences. Control region possessed several distinct characteristics, including: variable size, abundant tandem repetitions, and intra-genus conservation; and was useful in evolutionary and population genetic studies. The AGG codon reassignments were investigated between serine and lysine in the genera *Adelphocoris* and other cimicomorphans. Our analysis revealed correlated evolution between reassignments of the AGG codon and specific point mutations at the antidocons of *tRNA^Lys^* and *tRNA^Ser(AGN)^*. Phylogenetic analysis indicated that mitochondrial genome sequences were useful in resolving family level relationship of Cimicomorpha. Comparative evolutionary analysis of plant bug mitochondrial genomes allowed the identification of previously neglected coding genes or non-coding regions as potential molecular markers. The finding of the AGG codon reassignments between serine and lysine indicated the parallel evolution of the genetic code in Hemiptera mitochondrial genomes.

## Introduction

The Miridae (Hemiptera: Heteroptera: Cimicomorpha), or plant bugs, are one of the 20 most diverse families of insects and the largest family of true bugs belonging to the suborder Heteroptera, with approximately 11,000 described species in 1,200 genera [1], [2]. Plant bugs exhibit great morphological diversity and a wide range of food preferences and behaviors, including phytophagy, carnivory, and omnivory [1], [3]. Some mirid species exhibit significant economic impacts, e.g., some are pests of food and fiber crops, whereas others are beneficial species used as biological control agents [1], [4]. Field trials conducted over 10 years in northern China show that some plant bugs have progressively increased population sizes and acquired pest status in cotton and multiple other crops, in association with a regional increase in Bt cotton adoption [5].

Despite their economic and evolutionary importance, plant bugs are rarely recognized [1]. The application of DNA sequence data to mirid systematics has had minimal impact to date. Only a small amount of mitochondrial (16S rDNA and *COI*) and nuclear sequence data (18S and 28S rDNA) are used to study cimicomorphan relationships [6], [7] and the phylogeny of Miridae [2]. The use of DNA sequence data in species level studies has been nearly negligible [1], [8].

The mitochondrial (mt) genome is, to date, the most extensively studied genomic marker (s) in insects at genomic level [9]. In spite of an ongoing debate concerning their utility in phylogenetics [10]–[14], mt genomic studies have proven to be informative and insightful for phylogenetic [15]–[21] and phylogeographic studies [22]. This can be explained by conceptual advantages such as the simple genomic organization, (almost) unambiguous orthology of genes, and presence of rare genomic changes, including gene rearrangement and changes in the genetic code [23]–[25]. Up to now, only four complete mt genomes of plant bugs have been reported [26]–[29]. These four sequenced mt genomes have a large variation in genome size, ranging from 14,768 bp in *Apolygus lucorum* [27] to 17,027 bp in *Lygus lineolaris* [29], and most general genomic characteristics are conserved, e.g., gene content and gene arrangement. It is worth noting that the use of non-standard anticodons at two tRNAs, *tRNA^Lys^* and *tRNA^Ser(AGN)^*, is found in mt genome of *Adelphocoris fasciaticollis* [28].

In this study, a complete and four nearly complete mt genomes from three genera of plant bugs were sequenced. Finally, nine mt genomes representing two subfamilies and five genera of the Miridae and other 13 species from four cimicomorphan families were used in the comparative analysis to: 1) explore the molecular basis of the anticodon mutations of *tRNA^Lys^* and *tRNA^Ser(AGN)^* in the genus *Adelphocoris* and the evolution of the genetic code in Cimicomorpha and 2) assess the phylogenetic utility of mt genomic data at different taxonomic levels of Cimicomorpha and Miridae.

## Materials and Methods

### Ethics statement

No specific permits were required for the insects collected for this study. The insect specimens were collected from cotton fields by sweeping. The field collections did not involve endangered or protected species. The species sequenced in the family Miridae are common insects and are not included in the “List of Protected Animals in China”.

### Samples and DNA extraction

All samples used in this study were collected from cotton fields in China, and the collection information were provided in Table S1. Specimens were initially preserved in 100% ethanol in the field, and then conserved at −20°C for the long-term storage at the China Agricultural University (CAU). For each species, the genomic DNA was extracted from one adult’s muscle tissues of the thorax using the DNeasy DNA Extraction kit (Qiagen).

### PCR amplification and sequencing

For each species, mt genome was amplified by PCR in overlapping fragments with universal insect mt primers [24], and species-specific primers designed from sequenced fragments. All primers used in the present study were listed in Table S2. PCR and sequencing reactions were conducted following Li et al. [19], [30].

### Genome assembly and annotation

Sequence reads from the mt genome of each species were assembled into contigs with BioEdit 7.0.5.3 [31]. tRNA genes were identified with tRNAscan-SE 1.21 [32]. Some tRNA genes, which could not be identified by tRNAscan-SE, were determined by sequence similarity comparison with tRNA genes of other true bugs [26], [27], [33], [34]. Protein-coding genes (PCGs) and rRNA genes were identified by BLAST searches in GenBank and then confirmed by alignment with homologous genes from other true bugs [26], [27], [33], [34].

### Genomic analyses

At present, a total of 15 complete and seven nearly complete mt genome sequences of Cimicomorpha (Hemiptera: Heteroptera) including five plant bugs sequenced from present study were available in GenBank. Nucleotide composition of 15 complete mt genome sequences was calculated using Mega 5.0 [35]. AT- and GC-skew [36] were used to measure base compositional differences of mt genomes between the Miridae (plant bug) and other relative cimicomorphan families, e.g., Reduviidae (assassin bug), Nabidae (damsel bug) and Tingidae (lace bug). For each species of 22 cimicomorphans, we concatenated the 13 mt PCGs and used MEGA 5.0 to calculate 1) the overall nucleotide G+C% using all three codon positions, and 2) the frequency of amino acids encoded by GC-rich codons (G+A+R+P%).

The mt genetic code of each species was determined using GenDecoder v1.6 [37], [38]. This method has been proven to be a highly reliable prediction of genetic code [39] and it is basically dependent on the number of occurrences of each codon in conserved positions of the alignment. Due to the very low number of codon usage, GenDecoder predictions of AGG codons were carefully reviewed to identify dubious codon assignments and correct them based on aligned sequences of PCGs of nine plant bugs or 22 cimicomorphan insects.

### Phylogenetic analyses

A total of 24 species of heteropteran insects were used in phylogenetic analyses, including 22 cimicomorphans and two outgroup species from Pentatomomorpha. The cimicomorphan species were: a flower bug (Anthocoridae), a lace bug (Tingidae), five assassin bugs (Reduviidae), six damsel bugs (Nabidae) and nine plant bugs (Miridae). Details of the species used in this study were listed in Table 1.

**Table 1:** Species used in this study.

Infraorder	Family	Species	GenBank accession number	Reference
Pentatomomorpha	Largidae	*Physopelta gutta*	NC_012432	[40]
	Malcidae	*Malcus inconspicuus*	NC_012458	[40]
Cimicomorpha	Anthocoridae	*Orius niger* *	NC_012429	[40]
	Tingidae	*Corythucha ciliata*	NC_022922	[41]
	Reduviidae	*Agriosphodrus dohrni*	NC_015842	[33]
		*Sirthenea flavipes*	NC_020143	[42]
		*Triatoma dimidiata*	NC_002609	[43]
		*Valentia hoffmanni*	NC_012823	[44]
		*Oncocephalus breviscutum*	NC_022816	[45]
	Nabidae	*Alloeorhynchus bakeri*	NC_016432	[46]
		*Gorpis annulatus*	NC_019595	[34]
		*Gorpis humeralis*	NC_019593	[34]
		*Himacerus apterus* *	JF927831	[34]
		*Himacerus nodipes* *	JF927832	[34]
		*Nabis apicalis*	NC_019594	[34]
	Miridae	*Apolygus lucorum*	NC_023083	[27]
		*Lygus lineolaris*	NC_021975	[29]
		*Lygus rugulipennis* *	KJ170898	present study
		*Nesidiocoris tenuis*	NC_022677	[26]
		*Adelphocoris fasciaticollis*	KJ001714	[28]
		*Adelphocoris lineolatus*	KJ020286	present study
		*Adelphocoris nigritylus* *	KJ020287	present study
		*Adelphocoris suturalis* *	KJ020288	present study
		*Trigonotylus caelestialium* *	KJ170899	present study

*, nearly complete mt genome.

Sequences of 13 PCGs, two rRNAs and 19 tRNAs were used in phylogenetic analyses. Three tRNAs which were not amongst the commonly reported 22 tRNAs in most cimicomorphans, were excluded, i.e. *tRNA^Ile^*, *tRNA^Gln^* and *tRNA^Met^*. Each PCG was aligned individually based on codon-based multiple alignments by using the MAFFT algorithm within the TranslatorX [47] online platform. Poorly aligned sites were removed from the protein alignment before back-translate to nucleotides by using GBlocks within the TranslatorX with default settings. The sequences of tRNAs and rRNAs were aligned respectively using MXSCARNA [48], which is a computer-based alignment approach to consider the predicted secondary structure of noncoding RNA. Ambiguous positions in the alignment of RNAs were filtered using GBlocks v0.91b [49] with default settings.

Individual genes were concatenated using SequenceMatrix v1.7.8 [50]. Four datasets were assembled for phylogenetic analyses: 1) nucleotides of 13 PCGs, two rRNAs and 19 tRNAs (nt123RNA) with 13,393 residues; 2) nucleotides of 13 PCGs (nt123) with 10,506 residues; 3) nucleotides of two rRNAs and 19 tRNAs (RNA) with 2,887 residues, and 4) amino acids of 13 PCGs (AA) with 3,502 residues.

The optimal partition strategy and models of each dataset was selected by PartitionFinder v1.1.1 [51]. We created an input configuration file that contained pre-define partitions, e.g., 60 partitions for nt123RNA, 39 partitions for nt123, 21 partitions for RNA, and 13 partitions for AA. We used the ‘‘greedy’’ algorithm with branch lengths estimated as ‘‘unlinked’’ and Bayesian information criterion (BIC) to search for the best-fit scheme (Table S3).

We performed maximum likelihood (ML) and Bayesian inference (BI) using the best-fit partitioning schemes recommended by PartitionFinder (Table S3). ML analyses were conducted with RAxML 8.0.0 [52]. We used GTRGAMMAI model for nucleotide datasets and PROTGAMMAIMTART for amino acids. Node support was calculated by acquiring bootstrap values from heuristic searches of 1000 resampled datasets, using the rapid bootstrap feature (random seed value 12345) [53]. Bayesian analyses were carried out using MrBayes 3.2.2 [54]. Two simultaneous runs of 20 million generations were conducted for the datasets and trees were sampled every 1000 generations, with the first 25% discarded as burn-in. Stationarity was considered to be reached when the average standard deviation of split frequencies was below 0.01. All RAxML and MrBayes analyses were conducted in the CIPRES Science Gateway v3.3 [55].

## Results and Discussion

### General features of plant bug mt genomes

**Figure 1:**
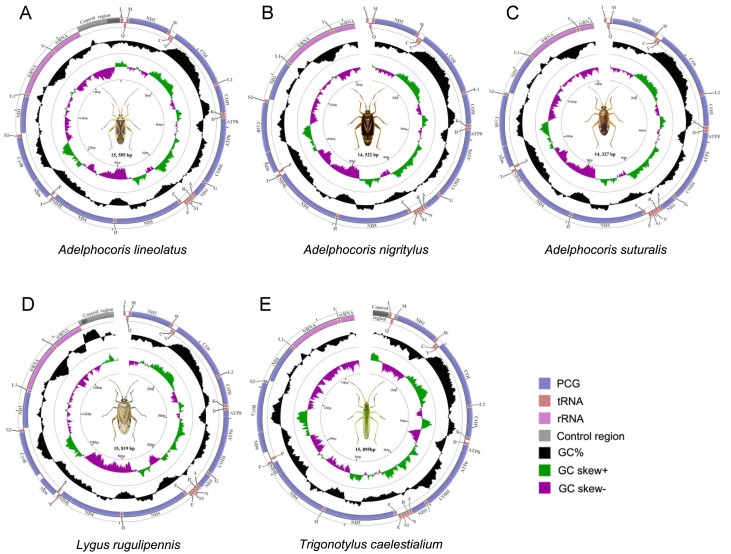
Mitochondrial genomes of five plant bugs sequenced in this study. Circular maps were drawn with CGView [56]. Arrows indicated the orientation of gene transcription. Abbreviations of gene names were: *ATP6* and *ATP8* for ATP synthase subunits 6 and 8, *COI*–*III* for cytochrome oxidase subunits 1–3, *CytB* for cytochrome *b*, *ND1–6* and *ND4L* for NADH dehydrogenase subunits 1–6 and 4L, *srRNA* and *lrRNA* for large and small rRNA subunits. tRNA genes were indicated with their one-letter corresponding amino acids (L1: CUN; L2: UUR; S1: AGN; S2: UCN). The GC content was plotted using a black sliding window, as the deviation from the average GC content of the entire sequence. GC-skew was plotted as the deviation from the average GC-skew of the entire sequence. The inner cycle indicated the location of genes in the mt genome.

In this study, mt genomes of five plant bugs sequenced for the first time (Figure 1), and five complete and four nearly complete mt genomes of plant bugs representing five genera and two subfamilies were compared. Five complete mt genomes were from *Nesidiocoris tenuis* [26], *Apolygus lucorum* [27], *Lygus lineolaris* [29], *Adelphocoris fasciaticollis* [28] and *Ad. lineolatus*, and four nearly complete mt genomes were from *Ad. suturalis*, *Ad. nigritylus*, *L. rugulipennis* and *Trigonotylus caelestialium*. The sequenced mt genomes of plant bugs were similar to mostly typical of other insect genomes and retained the ancestral insect mt genome arrangement [57]. Most of the size variation was due to differences in the control region, although some of the genomes had additional non-coding regions within the coding region. Some general characteristics of the genomes were given in Table S4.

All of the genomes examined showed base composition biases, the positive AT- and negative GC-skews (Figure S1), as is usually observed in insect mt genomes [58]. For PCGs, all species were characterized by mt coding sequences impoverished in G and C. Sequence of the lace bug, *Corythucha ciliata*, was extremely A + T-rich, as, to a lesser extent, were plant bugs, flower bugs and damsel bugs. Notably, the sequences of five assassin bugs had a less extreme nucleotide (and amino acid) composition (Figure S2A). As expected, the overall nucleotide composition and the proportion of the “GARP” amino acids were positive correlated (R^2^ = 0.89) [17]. Among nine plant bugs, species from the same genera shared a similar nucleotide (and amino acid) composition, e.g., the genera *Adelphocoris* and *Lygus* (Figure S2B).

### Non-coding regions

The non-coding regions of nine plant bugs were summarized in Table S5. The proportion of non-coding regions was high in *L. lineolaris* and *N. tenuis*, varying from 1.59 to 18.03%. Intra-genus species appeared to share the conserved sequence length and location of non-coding regions, e.g., three non-coding regions of *tRNA^Glu^* – *tRNA^Phe^* (10 to 21 bp), *tRNA^Pro^* – *ND6* (2 bp) and *ND6* – *CytB* (1 bp) were shared by four species from *Adelphocoris*; and two species from *Lygus* had three non-coding regions, *tRNA^Lys^* – *tRNA^Asp^* (2 bp), *tRNA^Pro^* – *ND6* (1 bp) and *ND6* – *CytB* (112 and 126 bp, with 75.4% identity).

In the typical insect mt genome, there are two sites where blocks of genes coded on different strands meet at their downstream ends [59], [60]. In *Drosophila melanogaster*, 16 bp non-coding sequences having significant sequence similarity are present at both sites. These sequences have been shown to be binding sites for a bidirectional transcription termination factor, DmTTF [59]. Alignments of the sequences of these two regions in plant bugs and *D. melanogaster* were shown in Figure S3.

Examination of the first site, between *tRNA^Glu^* and *tRNA^Phe^*, showed that this binding site was not completely conserved across Miridae and was absent from the genera, *Nesidiocoris*, *Apolygus, Lygus* and *Trigonotylus* (Figure S3A). Sequences identity between *Adelphocoris* and *D. melanogaster* was very low. This DmTTF binding site might not present in plant bugs and was absent as well from other insect orders [60], [61].

The second DmTTF binding site, between *tRNA^Ser(UCN)^* and *ND1*, is more widely conserved. Similar non-coding sequences are present at this site in other insect orders [60]–[63]. All of the sequences determined in this study had a sequence of identical length (7 bp) and with significant similarity to the DmTTF binding site (Figure S3B).

**Figure 2:**
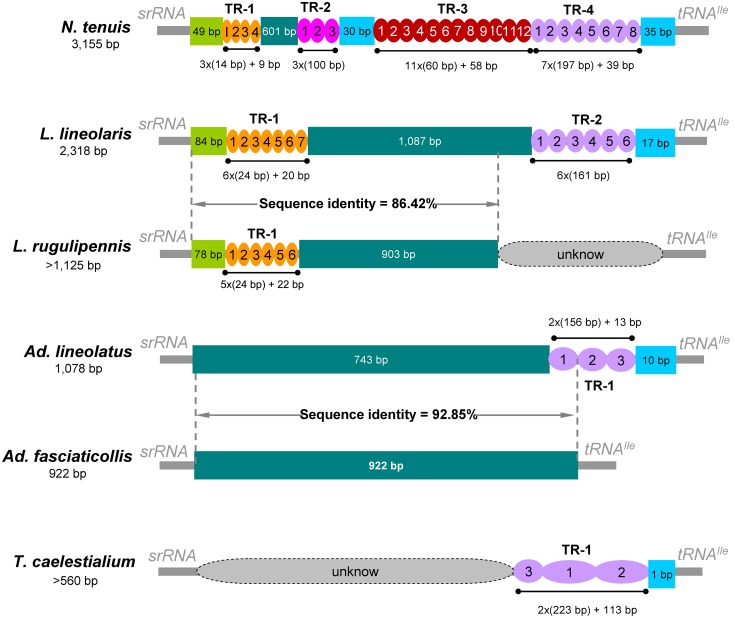
Organization of the control region in plant bug mitochondrial genomes. The location and copy number of tandem repeats were shown by colored oval with Arabic numerals inside. The remaining sequences of the control region were indicated by the colored rectangle. Intra-genus variations in sequences of control region were also shown in the genera *Adelphocoris* and *Lygus*. Mitochondrial control regions of *L. rugulipennis*, *Ad. lineolatus*, *Ad. fasciaticollis* and *T. caelestialium* were sequenced in this study.

The non-coding region located between *srRNA* and *tRNA^Ile^*, was annotated as control region (CR) by comparison with other insect mt genomes, including the presumed origin of replication and promoters for transcription initiation [64], [65]. Five of the nine sequences, where complete CRs were determined, were relatively variable, ranging from 228 bp in *Apolygus* to 3,155 bp in *Nesidiocoris* (Figure 2). There was no tandem repeat sequence in the CR of *Ap. lucorum* and *Ad. fasciaticollis*; but in other species, tandem repeat sequences were largely abundant. Four of the sequences include large tandem repeats present in two or more copies (Figure 2). The complete sequences of *N. tenuis* CR had four regions including tandem repeats: 1) a short 14 bp sequence tandemly repeated three times, with a partial forth (9 bp); 2) a 100 bp sequence tandemly repeated three times; 3) a 60 bp sequence tandemly repeated 11 times, with a partial twelfth (58 bp); and 4) a 197 bp sequence tandemly repeated seven times, with a partial eighth (39 bp). *L. lineolaris* had a short 24 bp sequence tandemly repeated six times, with a partial seventh (20 bp), and a 161 bp sequence present in six perfect copies. In *Ad. lineolatus*, there was a tandem repeat of a 156 bp sequence, present in two copies with a partial third (13 bp). Finally, two plant bugs, *T. caelestialium* and *L. rugulipennis*, with a partial CR sequence encountered in this study, had a 223 bp sequence tandemly repeated at least two times (two copies and a partial 113 bp sequence) and a short 24 bp sequence tandemly repeated five times with a partial sixth (22 bp), respectively.

CRs from different insect species always exhibit a very high level divergence [66]. Comparison of the nucleotide sequences of plant bug CRs (tandem repeats were removed) showed that this region appeared to be heterogeneous in the inter-genus level (identity = 26.66%), but was homogeneous in the intra-genus level, e.g., two species in *Lygus* (identity = 86.42%) and two species in *Adelphocoris* (identity = 92.85%) (Figure 2).

As we have seen, plant bug mt CRs possess several distinct structural and evolutionary characteristics, including: variable size, abundant tandem repetition, intra-genus conservation, etc. These characteristics have important implications for the usefulness of this region in evolutionary and population genetic studies of the Miridae.

### Correlated evolution of the point mutation at anticodon of tRNA genes and the AGG codon reassignments between serine and lysine

The tRNAs coded in four *Adelphocoris* species could be folded into a classical clover-leaf secondary structure. However, in other plant bugs, *tRNA^Ser(AGN)^* evidently lacked DHU stem-loop structures. Structures similar to these have been previously observed in many other true bugs [19], [33], [34], [40]. The sequences identity of the tRNAs from five plant bug genera were given in Figure S4. The regions including the entire anticodon arms and DHU stems were very well conserved across the plant bugs in all 22 tRNAs. Most of the variations were within regions of the DHU loops, TψC arms and variable loop, including both nucleotide substitutions and indels. Intra-genus species presented a high sequence identity (Figures S5, S6).

Most tRNAs used the standard anticodon in plant bugs and other cimicomorphans, with only two exceptions. These exceptions were that *tRNA^Lys^* was predicted to have anticodon UUU, and *tRNA^Ser(AGN)^* had the anticodon UCU in the four species of the genus *Adelphocoris* (Figures S4, S5). The genetic code provides the translation table between the DNA and protein languages by establishing correspondences between codons and amino acids [67]. Although the genetic code is nearly universal, several variants of this code have been described in a wide range of nuclear and organellar systems, especially in metazoan mitochondria, where more than 10 variants have been described [39], [67]–[70]. A new genetic code that translate the codon AGG as lysine (Lys) instead of serine (Ser) (as in the invertebrate mitochondrial genetic code) has been found in several arthropods [39], [67], and the specific point mutations at the anticodons might explain the recurrence of the AGG reassignments [39].

**Figure 3:**
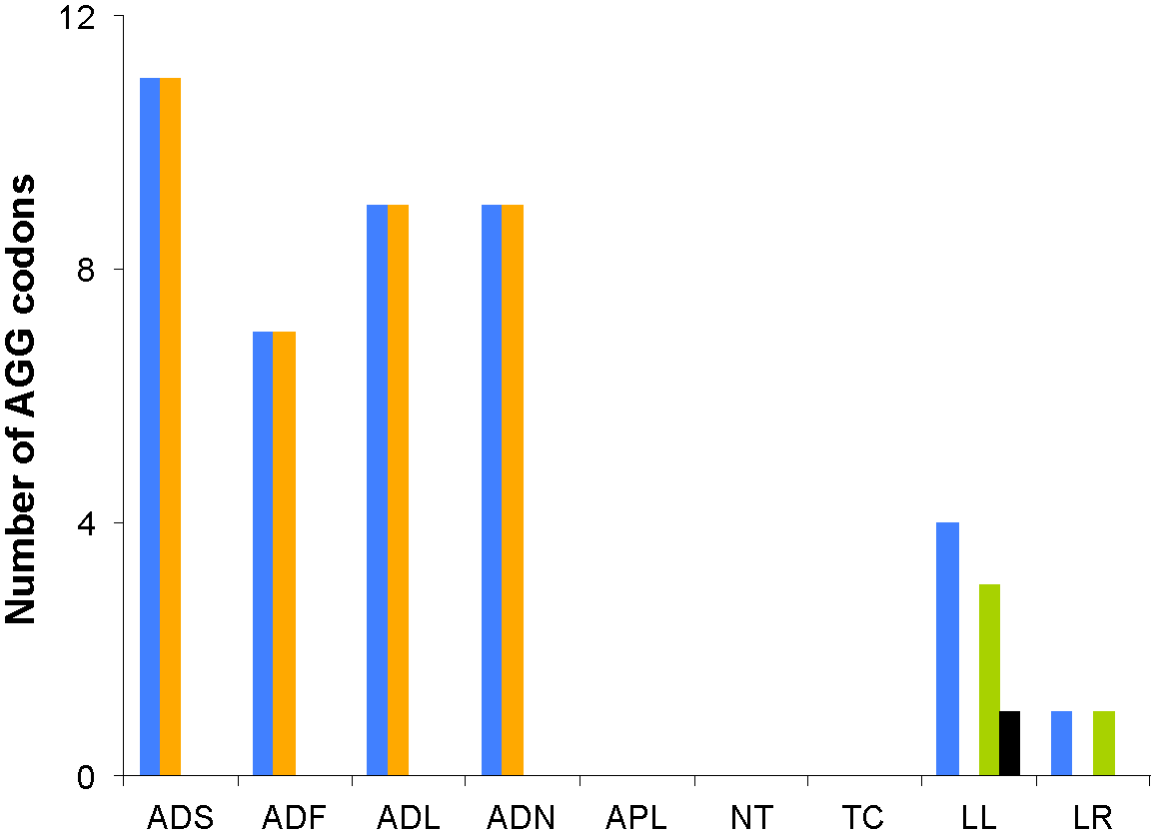
Usage of the AGG codons in nine plant bug mitochondrial genomes. Blue, total number of the AGG codons; orange, number of the AGG codons predicted to translate as Ser; green, number of the AGG codons predicted to translate as Lys; black, number of unpredicted AGG codons. ADS, *Ad. suturalis*; ADF, *Ad. fasciaticollis*; ADL, *Ad. lineolatus*; ADN, *Ad. nigritylus*; APL, *Ap. lucorum*; NT, *N. tenuis*; TC, *T. caelestialium*; LL, *L. lineolaris*; LR, *L. rugulipennis*.

The point mutations at the anticodons of both *tRNA^Lys^* and *tRNA^Ser(AGN)^* were investigated in the plant bug genus *Adelphocoris*. Codon usage analysis of nine plant bugs showed that only two genera *Adelphocoris* and *Lygus* made use of AGG codon, especially a high usage in *Adelphocoris* (Figure 3). We predicted amino acid assignments for the AGG codon by using GenDecoder and based on aligned sequences of PCGs of nine plant bugs. According to our result, four species from *Adelphocoris* translate AGG as Ser and two species form *Lygus* translate it as Lys (Figures 3, S7). Obviously, the AGG codon was reassigned in mt genome of plant bugs.

**Figure 4:**
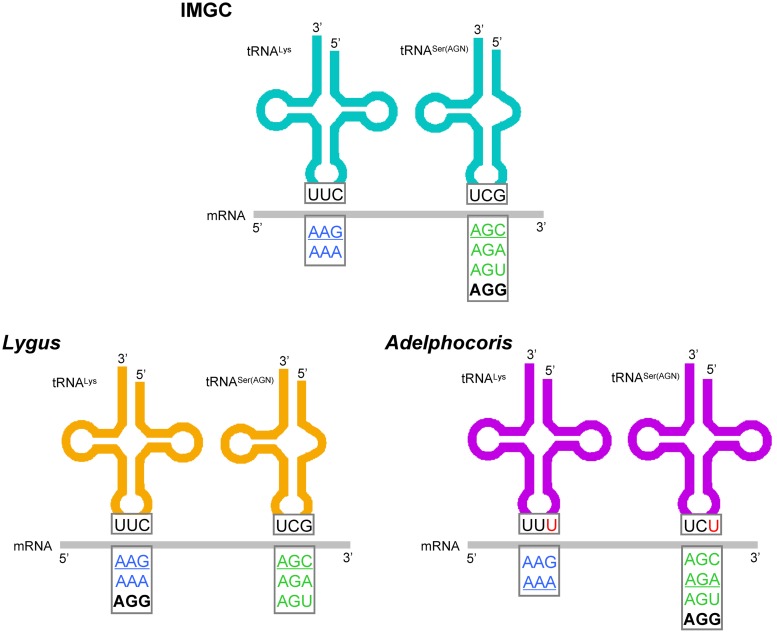
The molecules of *tRNA^Ser(AGN)^* and *tRNA^Lys^* in plant bug mitochondrial genomes and the AGG codon reassignments. The *tRNA^Ser(AGN)^* and *tRNA^Lys^* anticodons in genus decoding as Ser or Lys, as well as the predicated translation of AGN and AAR mRNA-codons, were shown. IMGC, Invertebrate Mitochondrial Genetic Code. Anticodons were depicted in 3′ to 5′ sense, e.g., UUC in *tRNA^Lys^* corresponded to the anticodon CUU in standard notation. The point mutations of anticodon in the genus *Adelphocoris* were highlighted by red color.

We also found that mutations at anticodons in both *tRNA^Lys^* and *tRNA^Ser(AGN)^* were correlated with the AGG codon reassignment (Figure 4) [39]. In the case of *tRNA^Ser(AGN)^*, anticodon mutations were highly diagnostic for AGG codon. All *Adelphocoris* species predicted to decode AGG as Ser changed the typical anticodon GCU of the *tRNA^Ser(AGN)^* to UCU (Figure 4). The anticodon of *tRNA^Lys^* was also strongly associated with the meaning of AGG. In nine plant bugs, five species have a *tRNA^Lys^* with the anticodon CUU, whereas other four species form *Adelphocoris* have UUU. Two species from *Lygus* predicted to decode AGG as Lys have the CUU anticodon, although three species from *Nesidiocoris*, *Apolygus*, and *Trigonotylus* that have the CUU anticodon do not use AGG (Figures 3,4).

The number of AGG codon tends to be higher when AGG is translated as Ser. This is possibly caused by the better ability of the mutated *tRNA^Ser(AGN)^* (UCU) to recognize the AGG codon compared to the *tRNA^Lys^* (CUU), which requires a wobble pairing at the second position of the codon. In fact, as previously suggested [39], *tRNA^Ser(AGN)^* seems to have a dominant phenotype compared to *tRNA^Lys^*. The strong association between mutation at the *tRNA^Ser(AGN)^* anticodon and translation of AGG suggests that this rather simple molecular change could explain the reassignment of AGG between Lys and Ser (Figure 4) [39].

### Phylogenetic relationships among five cimicomorphan families and the evolution of AGG codon reassignments

Cimicomorpha, which consists of 17 families representing more than 20,000 species, is the largest infraorder in Heteroptera [7], [71]. Members of this group show a wide range of adaptations to diverse habitats and life-history strategies [3], including predation and blood feeding in the Reduviidae and Cimicidae, mostly plant feeding in the Miridae and Tingidae. This grouping contains the two largest families of the true bugs, Miridae and Reduviidae. The family level relationship of Cimicomorpha, however, has been controversial for decades. Schuh and Štys [72] firstly analyzed the cimicomorphan relationships in a cladistic framework. The major conclusions of their analyses are the sister-group relationship of Reduvioidea (Reduviidae+Pachynomidae) to the remaining Cimicomorpha, the paraphyly of Anthocoridae *sensu lato*, and Miroidea to comprise Thaumastocoridae + (Tingidae + Miridae). These hypotheses are further supported by the combined morphological and molecular analysis [7]. Analyses of nuclear 18S rDNA, 28S rDNA and mitochondrial 16S rDNA sequences, however, indicate that Miroidea to be polyphyletic, with Tingidae repeatedly recovered as the sister group to all remaining Cimicomorpha; Reduviidae to be monophyletic but never recovered in a basal position; Cimiciformes (Naboidea + Cimicoidea) to be paraphyletic; and Cimicoidea to be monophyletic [6].

**Figure 5:**
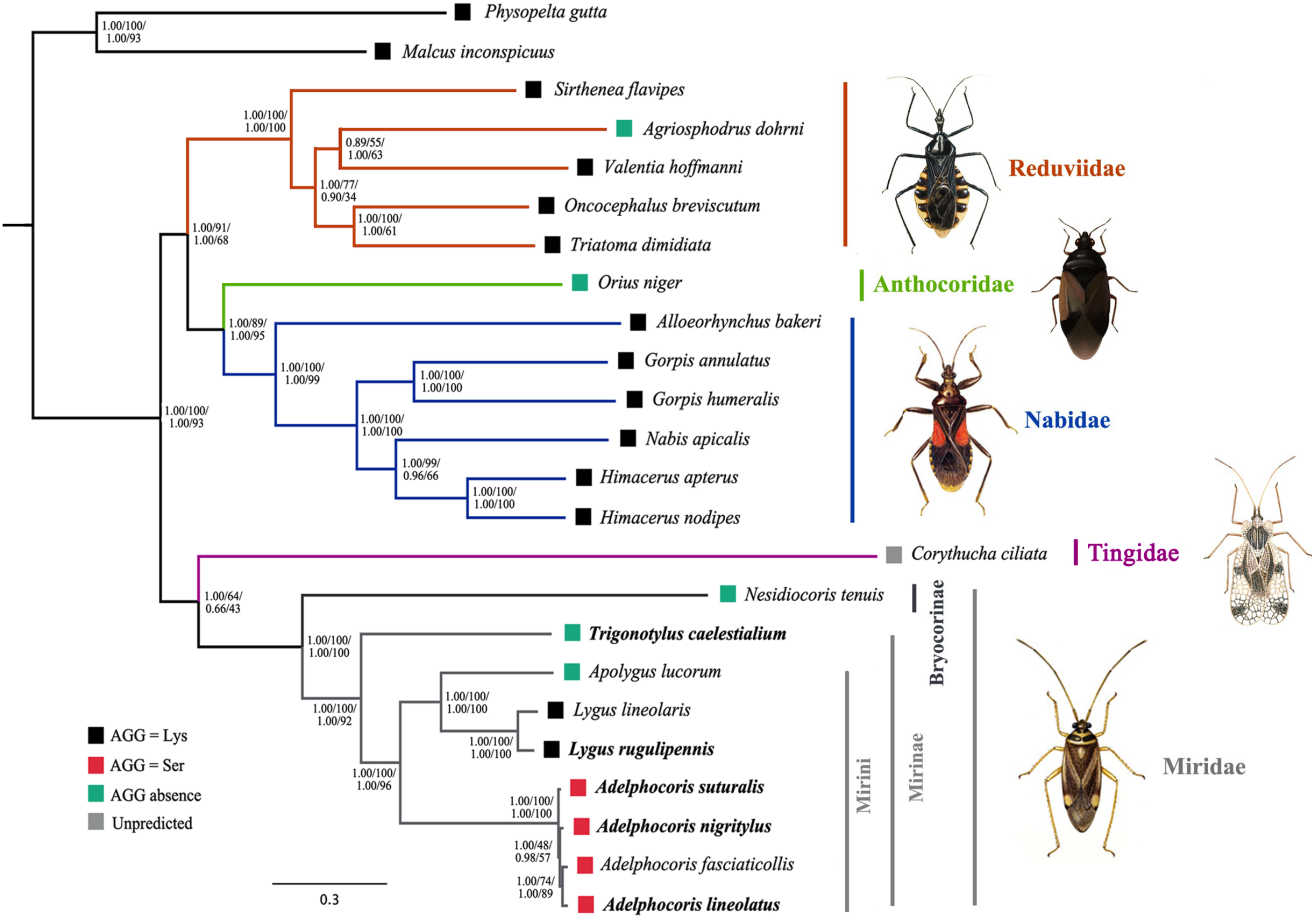
Phylogenetic relationships among five cimicomorphan families inferred from mitochondrial genome sequences. Numbers close to the branching points were Bayesian posterior probabilities and ML bootstrap support values. Numbers from left to right were from nt123RNA and RNA datasets respectively. To the right of the tree, the predicted translation of the AGG codon was shown for each taxon. The newly sequenced species were highlighted in bold.

We tested the phylogenetic relationships among the five families of the Cimicomorpha with 22 mt genome sequences available to date. The topologies of the phylogenetic trees inferred from two methods (BI and ML) and four nucleotide datasets (nt123RNA, nt123, RNA and AA) were almost identical (Figures 5, S8, S9): 1) five assassin bugs (Reduviidae) were monophyletic, so were six damsel bugs (Nabidae) and nine plant bugs (Miridae); 2) Miroidea (Miridae + Tingidae) were monophyletic; 3) Reduviidae was the sister-group to Anthocoridae and Nabidae (Figures 5, S8) or Nabidae (AA-ML, Figure S9), rather than the sister group to the remaining cimicomorphan families; and 4) in the family Miridae, eight plant bugs from the subfamily Mirinae was monophyletic, so were the tribe Mirini. Many groups from different taxonomic levels of Cimicomorpha were well recovered in our results, from superfamily to genus. Although the phylogenetic analyses based on the current taxon was limited to inferring the family level relationships of Cimicomorpha, it still had important implications for the usefulness of mt genome sequence in evolutionary and phylogenetic studies of Cimicomorpha and Miridae.

In order to better understanding the evolution of AGG codon reassignments, predicted amino acid assignments for the AGG codon of all analyzed mt genomes were mapped onto the reconstructed phylogeny of Cimicomorpha (Figure 5). Out of the 22 species, five made no use of the AGG codon. The AGG codon was predicted to translate as Lys in 14 species and as Ser only in four plant bugs from *Adelphocoris*. Abascal et al. (2012) analyzed the use of the AGG codon of more than 40 complete mt genomes of Hemiptera and found the significant discrepancy between two main lineages, Heteroptera that mostly translate AGG as Lys and non-heteropterans that mostly translate it as Ser [67]. The evolutionary pattern with AGG codon use in Cimicomorpha coincided with the Heteroptera that mostly translate AGG as Lys. This suggested that the ancestors of Heteroptera and Cimicomorpha might be predicted as AGG = Lys. Considering the AGG codon was predicted to translate as Ser in *Adelphocoris*, our results also indicated the parallel evolution of AGG codon reassignments between serine and lysine in Hemiptera mt genomes.

## Conclusions

With five newly sequenced mt genomes from the family Miridae, we presented the first comparative analysis of these plant bug mt genomes. Our results showed that gene content, gene arrangement, base composition and sequence of DmTTF binding site were conserved among plant bug mt genomes. Control region possessed several distinct characteristics, including: variable size, abundant tandem repetition, and intra-genus conservation, and was useful in evolutionary and population genetic studies of the Miridae. In general, our phylogenetic analysis also indicated that mt genome sequences were useful in resolving family level relationship of Cimicomorpha. More complete sampling from the family level in the future study should help clarify many of the outstanding questions of cimicomorphan phylogeny.

Before this study, shifts between alternative genetic codes have been known to be quite common within arthropod main lineages [39], [67]. Our analyses showed that the AGG codon was reassigned from Lys to Ser in the genera *Adelphocoris*, and revealed correlated evolution between reassignments of the AGG codon and specific point mutations at anticodon of *tRNA^Lys^* and *tRNA^Ser(AGN)^*. We investigated the evolution of the genetic code in the cimicomorphans and found the parallel evolution of AGG codon reassignments between serine and lysine in Hemiptera.

## Supporting Information

Figure S1 **Nucleotide compositional bias across 15 complete mitochondrial genomes from four cimicomorphan families.** Measured in bp percentage (X-axis) and level of nucleotide skew (Y-axis). Values were calculated on J-strands for full length of mt genomes. Triangle, AT-skews; circle, GC-skews. Four cimicomorphan families were shown by different colors: orange, Reduviidae (assassin bug); black, Nabidae (damsel bug); green, Tingidae (lace bug); and blue, Miridae (plant bug).
(TIFF)

Figure S2 **Compositional properties of mitochondrial coding sequences.** A, among five cimicomorphan families; B, among nine plant bugs. The G+C content of three codon positions in the concatenated alignment of PCGs was plotted against the percentage of amino acids encoded by G- and C-rich codons (glycine, alanine, arginine, and proline [G+A+R+P]). Values were averaged for some families, with SDs indicated.
(TIFF)

Figure S3 **Sequence alignments of two DmTTF binding sites between plant bugs and** ***Drosophila melanogaster*** **.** A, the DmTTF binding site between *tRNA^Glu^* and *tRNA^Phe^*; B, the DmTTF binding site between *tRNA^Ser (UCN)^* and *ND1*.
(TIFF)

Figure S4 **Inferred secondary structure of tRNA families in nine plant bug mitochondrial genomes.** The nucleotide substitution pattern for each tRNA family was modeled using as reference the structure determined for *Ad. fasciaticollis*. The identical nucleotides were shown by grey circles. Variations of nucleotides were highlighted by blue (sequence identity >60%) and red (sequence identity <60%) circles. The tRNAs were labeled with the abbreviations of their corresponding amino acids. Inferred Watson-Crick bonds were illustrated by lines, whereas GU bonds were illustrated by dots.
(TIF)

Figure S5 **Inferred secondary structure of tRNA families in four plant bug mitochondrial genomes from the genus** ***Adelphocoris*** **.** The nucleotide substitution pattern for each tRNA family was modeled using as reference the structure determined for *Ad. fasciaticollis*. The identical nucleotides were shown by grey circles. Nucleotide mutations were highlighted by blue circles. The tRNAs were labeled with the abbreviations of their corresponding amino acids. Inferred Watson-Crick bonds were illustrated by lines, whereas GU bonds were illustrated by dots.
(TIF)

Figure S6 **Inferred secondary structure of tRNA families in two plant bug mitochondrial genomes from the genus** ***Lygus*** **.** The nucleotide substitution pattern for each tRNA family was modeled using as reference the structure determined for *L. rugulipennis*. The identical nucleotides were shown by grey circles. Nucleotide mutations were highlighted by blue circles. The tRNAs were labeled with the abbreviations of their corresponding amino acids. Inferred Watson-Crick bonds were illustrated by lines, whereas GU bonds were illustrated by dots.
(TIF)

Figure S7 **Predicted amino acid assignments for the AGG codon based on the alignment sequences of PCGs of nine plant bugs.** The homologous positions including AGG codon were extracted from the alignment sequences of PCGs. The standard genetic codes were highlighted by different background colors: green for Lys, orange for Ser; and grey for other amino acids. The most frequent amino acid was then predicted to be the translation of the AGG codon and listed at the headline of the table. ? indicated that the AGG codon was defined as “unpredicted” due to the codon position was highly variable.
(TIFF)

Figure S8 **Phylogeny of Cimicomorpha produced from nt123 (BI and ML) and AA (BI).** Numbers close to the branching points were Bayesian posterior probabilities and ML bootstrap support values. Numbers from left to right were from nt123-BI, nt123-ML and RNA-BI respectively. The newly sequenced species were highlighted in bold.
(TIF)

Figure S9 **Phylogeny of Cimicomorpha produced from AA (ML).** Numbers close to the branching points were ML bootstrap support values. The newly sequenced species were highlighted in bold.
(TIF)

Table S1 Collection information of plant bugs sequenced in this study.
(DOCX)

Table S2 Primers used in this study.
(DOCX)

Table S3 The best partitioning scheme selected by PartitionFinder for different datasets.
(DOCX)

Table S4 Structural features of plant bug mitochondrial genomes.
(DOC)

Table S5 Statistics on non-coding sequences in plant bug mitochondrial genomes.
(DOC)
