# Supplementary figures and images for: Comparative Mitogenomics of Plant Bugs (Hemiptera: Miridae): Identifying the AGG Codon Reassignments between Serine and Lysine
Source: PLoS One. 2014 Jul 2;9(7):e101375. doi: 10.1371/journal.pone.0101375 (PMC4079613; doi:10.1371/journal.pone.0101375)

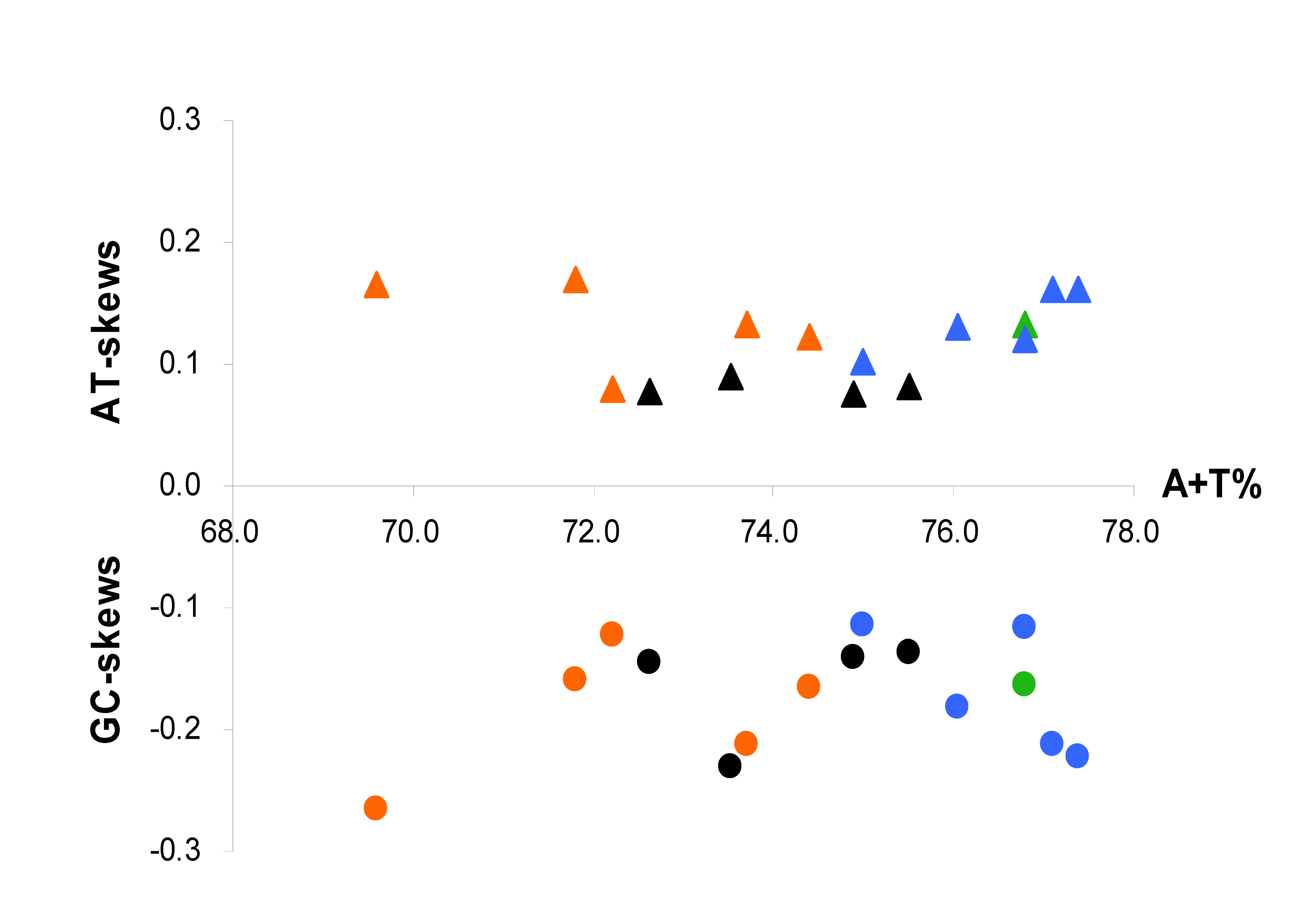

Supplement: Figure S1 — Nucleotide compositional bias across 15 complete mitochondrial genomes from four cimicomorphan families. Measured in bp percentage (X-axis) and level of nucleotide skew (Y-axis). Values were calculated on J-strands for full length of mt genomes. Triangle, AT-skews; circle, GC-skews. Four cimicomorphan families were shown by different colors: orange, Reduviidae (assassin bug); black, Nabidae (damsel bug); green, Tingidae (lace bug); and blue, Miridae (plant bug). (TIFF) [file pone.0101375.s001.tiff]

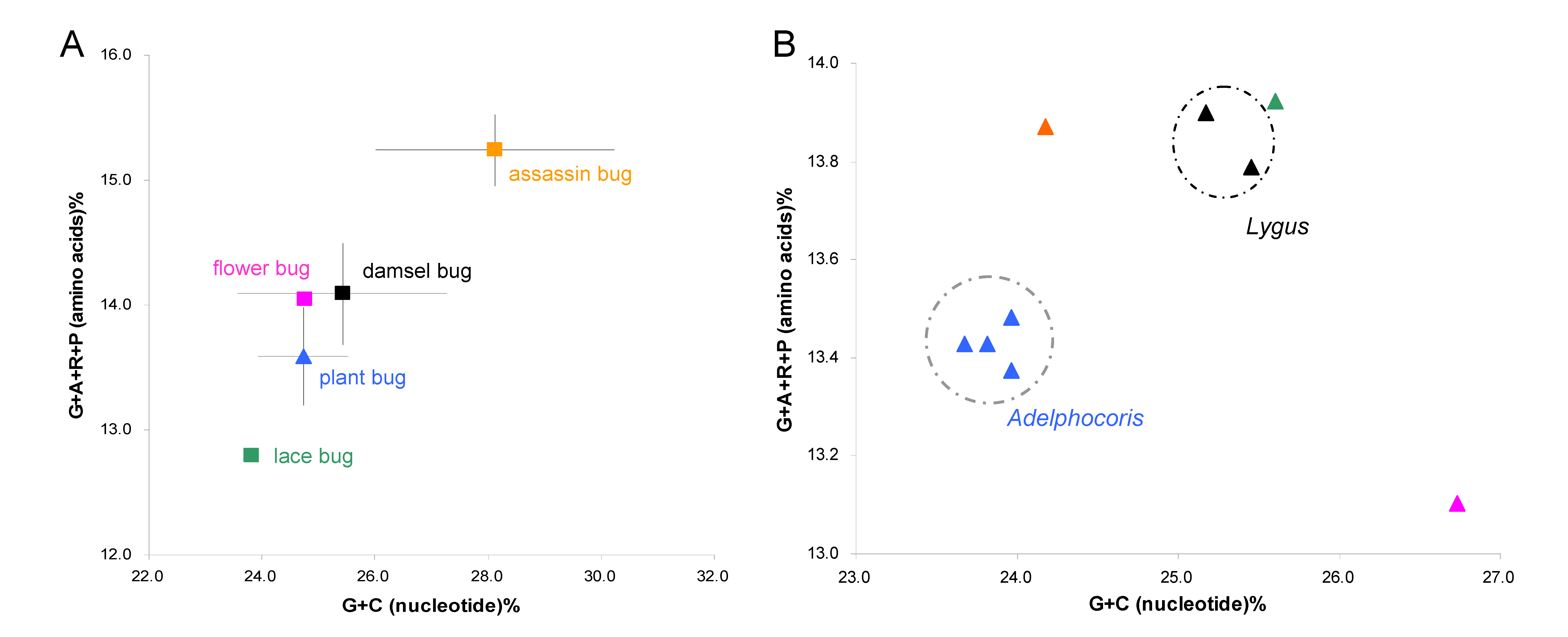

Supplement: Figure S2 — Compositional properties of mitochondrial coding sequences. A, among five cimicomorphan families; B, among nine plant bugs. The G+C content of three codon positions in the concatenated alignment of PCGs was plotted against the percentage of amino acids encoded by G- and C-rich codons (glycine, alanine, arginine, and proline [G+A+R+P]). Values were averaged for some families, with SDs indicated. (TIFF) [file pone.0101375.s002.tiff]

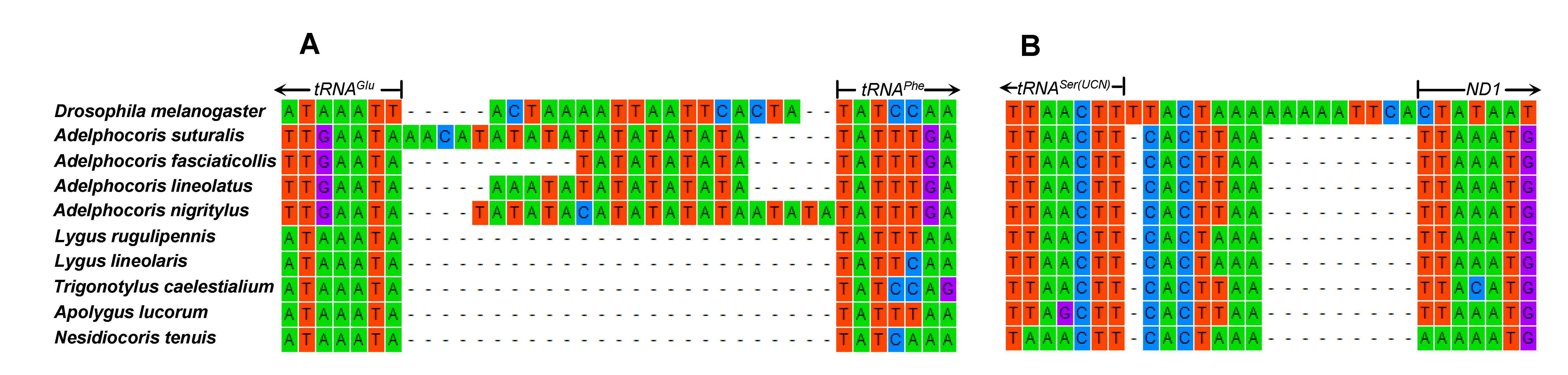

Supplement: Figure S3 — Sequence alignments of two DmTTF binding sites between plant bugs and Drosophila melanogaster . A, the DmTTF binding site between tRNAGlu and tRNAPhe; B, the DmTTF binding site between tRNASer (UCN) and ND1. (TIFF) [file pone.0101375.s003.tiff]

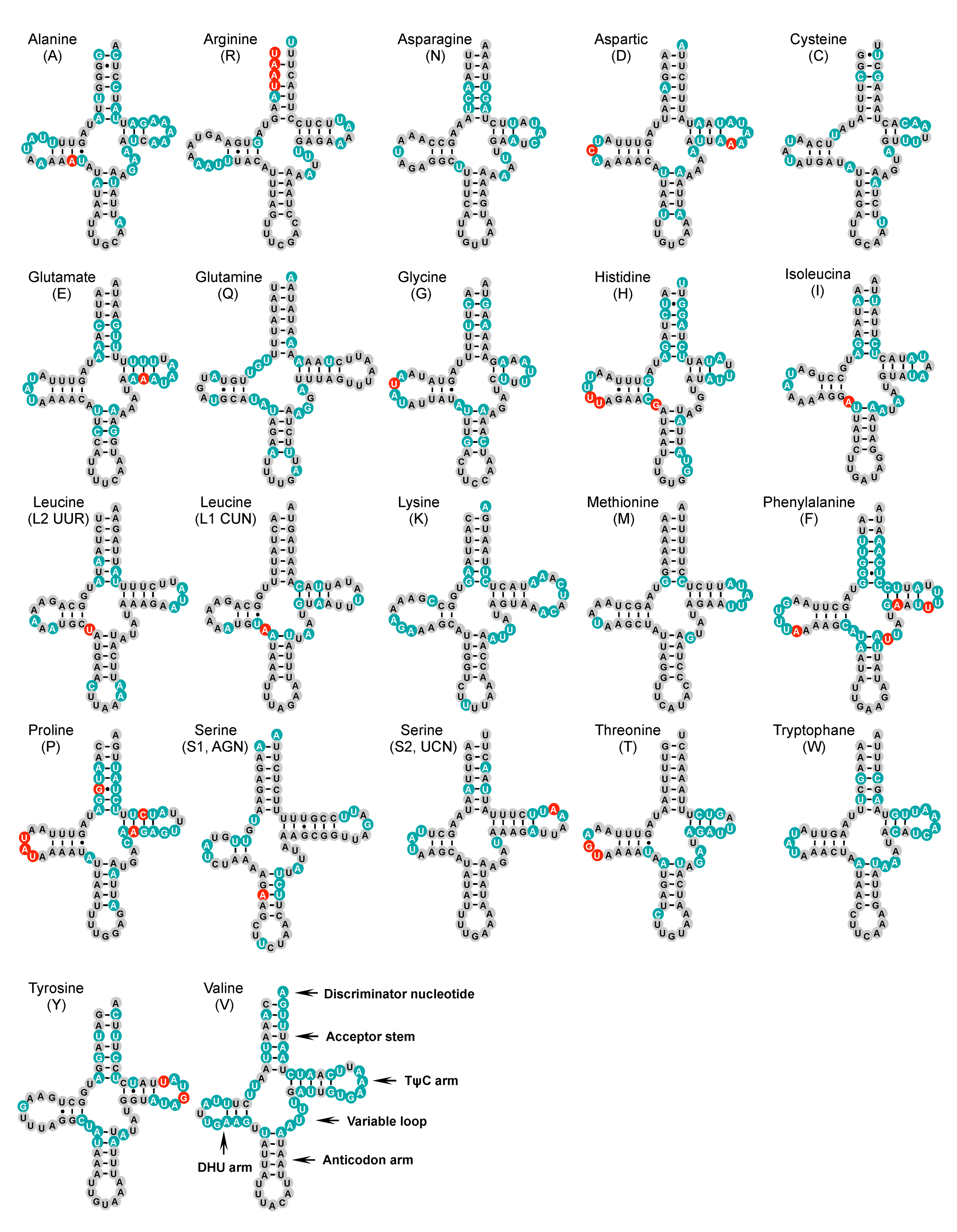

Supplement: Figure S4 — Inferred secondary structure of tRNA families in nine plant bug mitochondrial genomes. The nucleotide substitution pattern for each tRNA family was modeled using as reference the structure determined for Ad. fasciaticollis. The identical nucleotides were shown by grey circles. Variations of nucleotides were highlighted by blue (sequence identity >60%) and red (sequence identity <60%) circles. The tRNAs were labeled with the abbreviations of their corresponding amino acids. Inferred Watson-Crick bonds were illustrated by lines, whereas GU bonds were illustrated by dots. (TIF) [file pone.0101375.s004.tif]

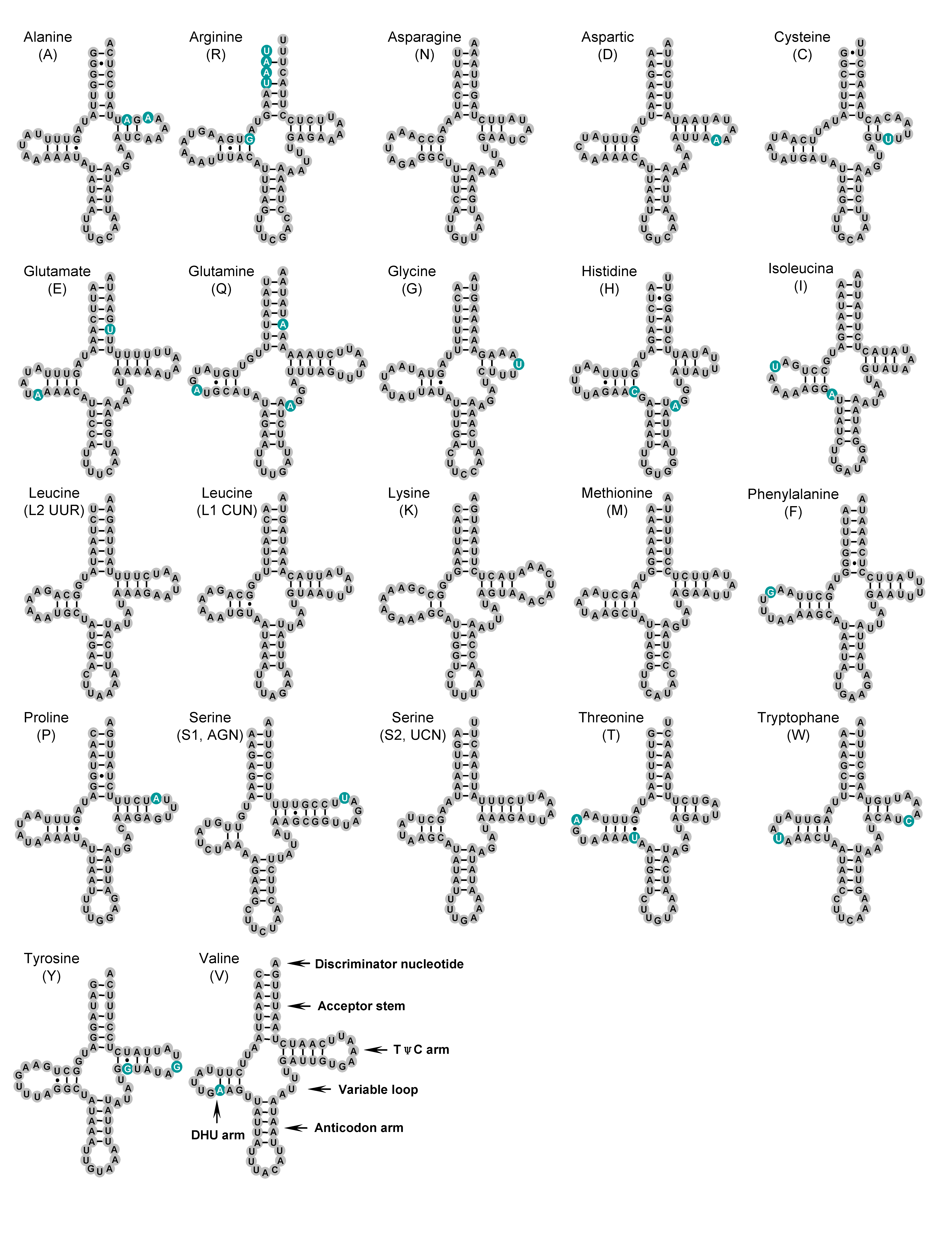

Supplement: Figure S5 — Inferred secondary structure of tRNA families in four plant bug mitochondrial genomes from the genus Adelphocoris . The nucleotide substitution pattern for each tRNA family was modeled using as reference the structure determined for Ad. fasciaticollis. The identical nucleotides were shown by grey circles. Nucleotide mutations were highlighted by blue circles. The tRNAs were labeled with the abbreviations of their corresponding amino acids. Inferred Watson-Crick bonds were illustrated by lines, whereas GU bonds were illustrated by dots. (TIF) [file pone.0101375.s005.tif]

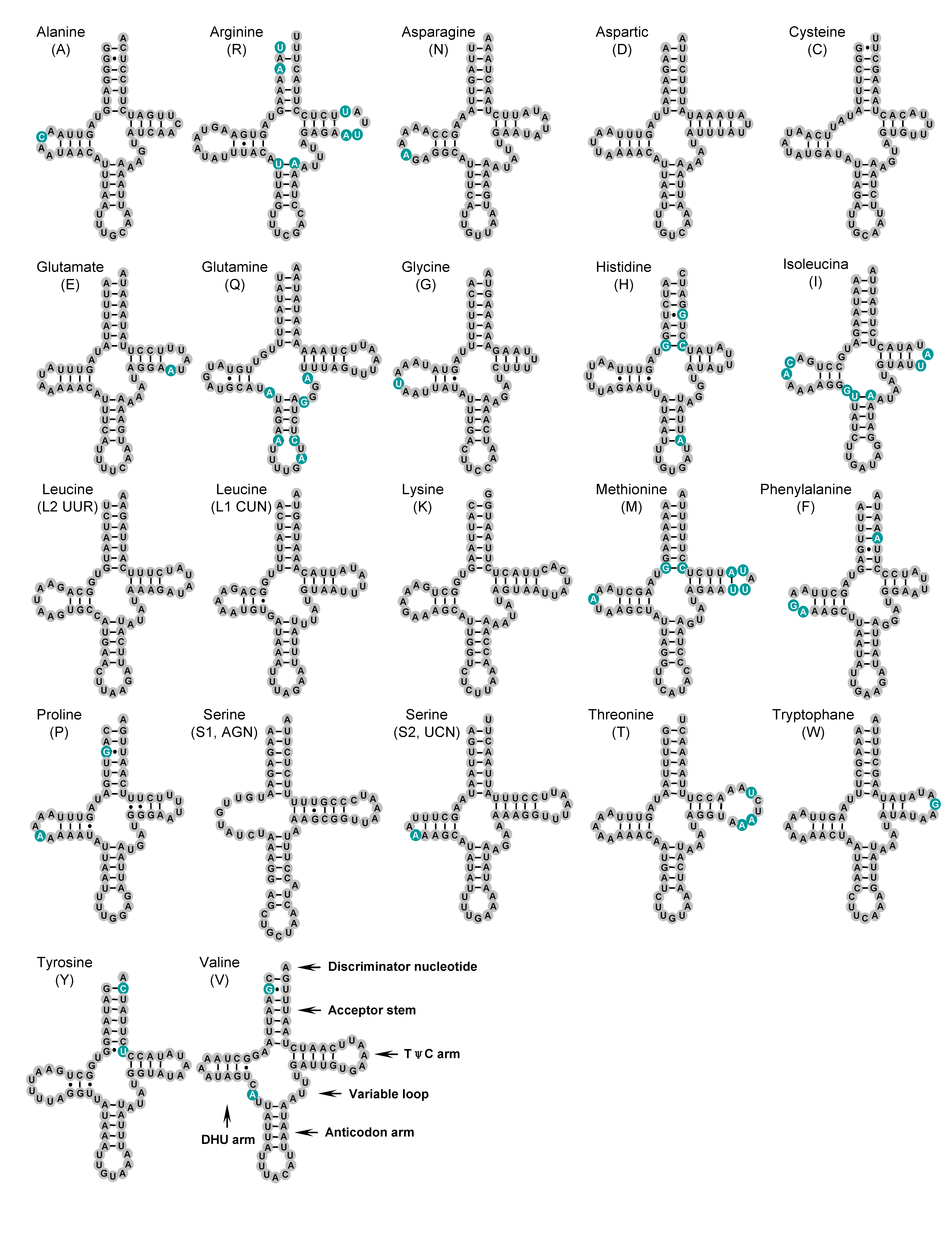

Supplement: Figure S6 — Inferred secondary structure of tRNA families in two plant bug mitochondrial genomes from the genus Lygus . The nucleotide substitution pattern for each tRNA family was modeled using as reference the structure determined for L. rugulipennis. The identical nucleotides were shown by grey circles. Nucleotide mutations were highlighted by blue circles. The tRNAs were labeled with the abbreviations of their corresponding amino acids. Inferred Watson-Crick bonds were illustrated by lines, whereas GU bonds were illustrated by dots. (TIF) [file pone.0101375.s006.tif]

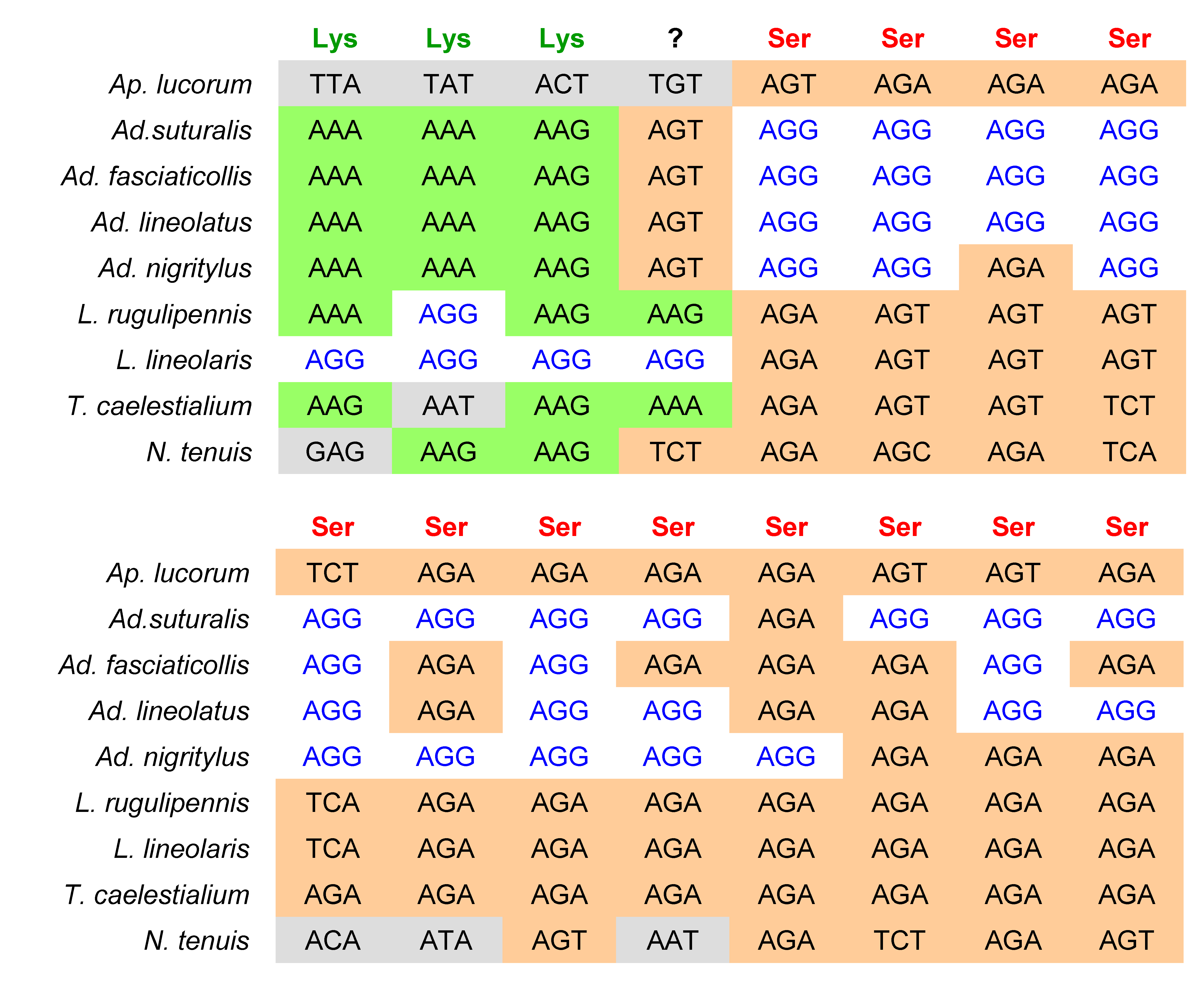

Supplement: Figure S7 — Predicted amino acid assignments for the AGG codon based on the alignment sequences of PCGs of nine plant bugs. The homologous positions including AGG codon were extracted from the alignment sequences of PCGs. The standard genetic codes were highlighted by different background colors: green for Lys, orange for Ser; and grey for other amino acids. The most frequent amino acid was then predicted to be the translation of the AGG codon and listed at the headline of the table. ? indicated that the AGG codon was defined as “unpredicted” due to the codon position was highly variable. (TIFF) [file pone.0101375.s007.tiff]

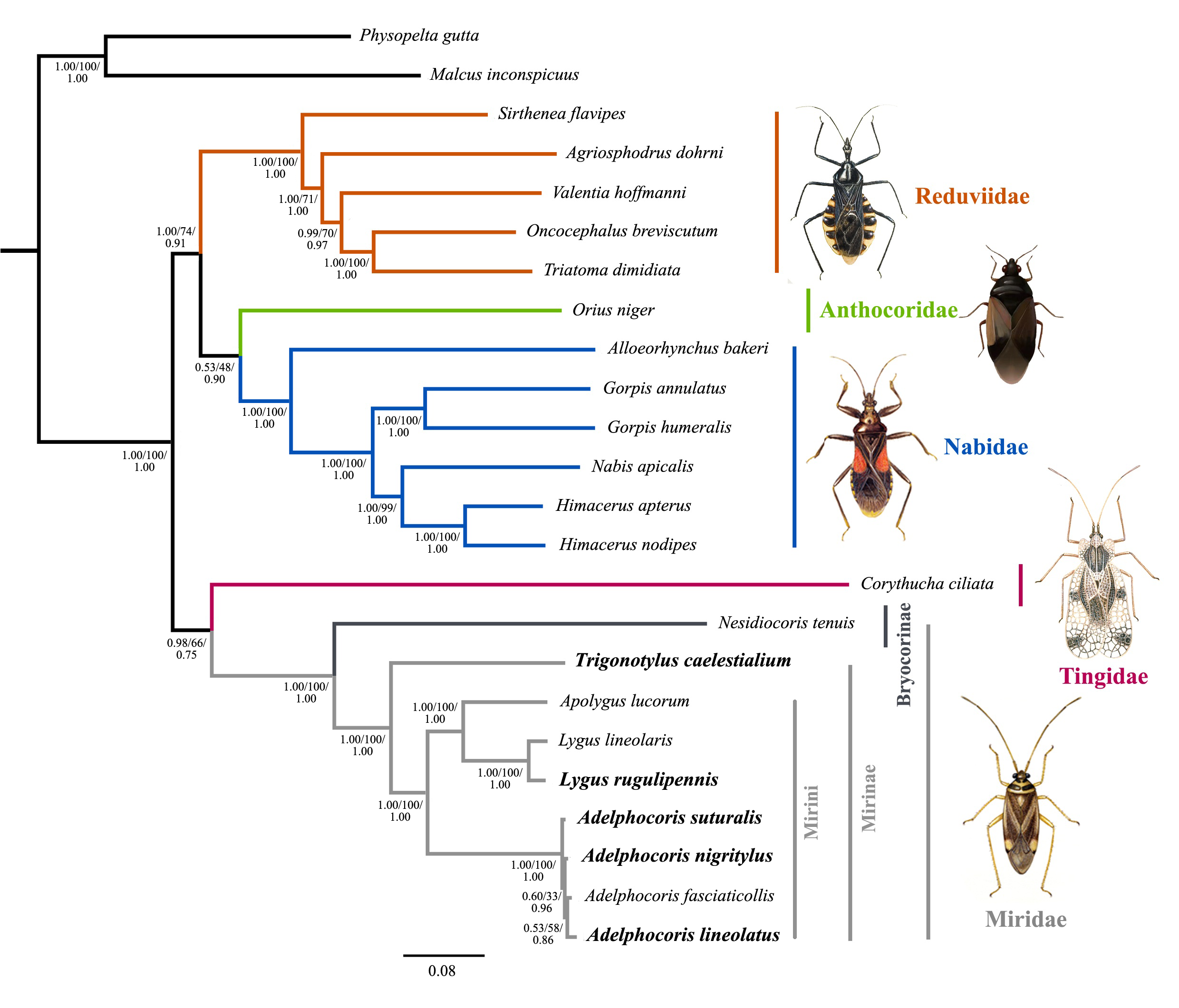

Supplement: Figure S8 — Phylogeny of Cimicomorpha produced from nt123 (BI and ML) and AA (BI). Numbers close to the branching points were Bayesian posterior probabilities and ML bootstrap support values. Numbers from left to right were from nt123-BI, nt123-ML and RNA-BI respectively. The newly sequenced species were highlighted in bold. (TIF) [file pone.0101375.s008.tif]

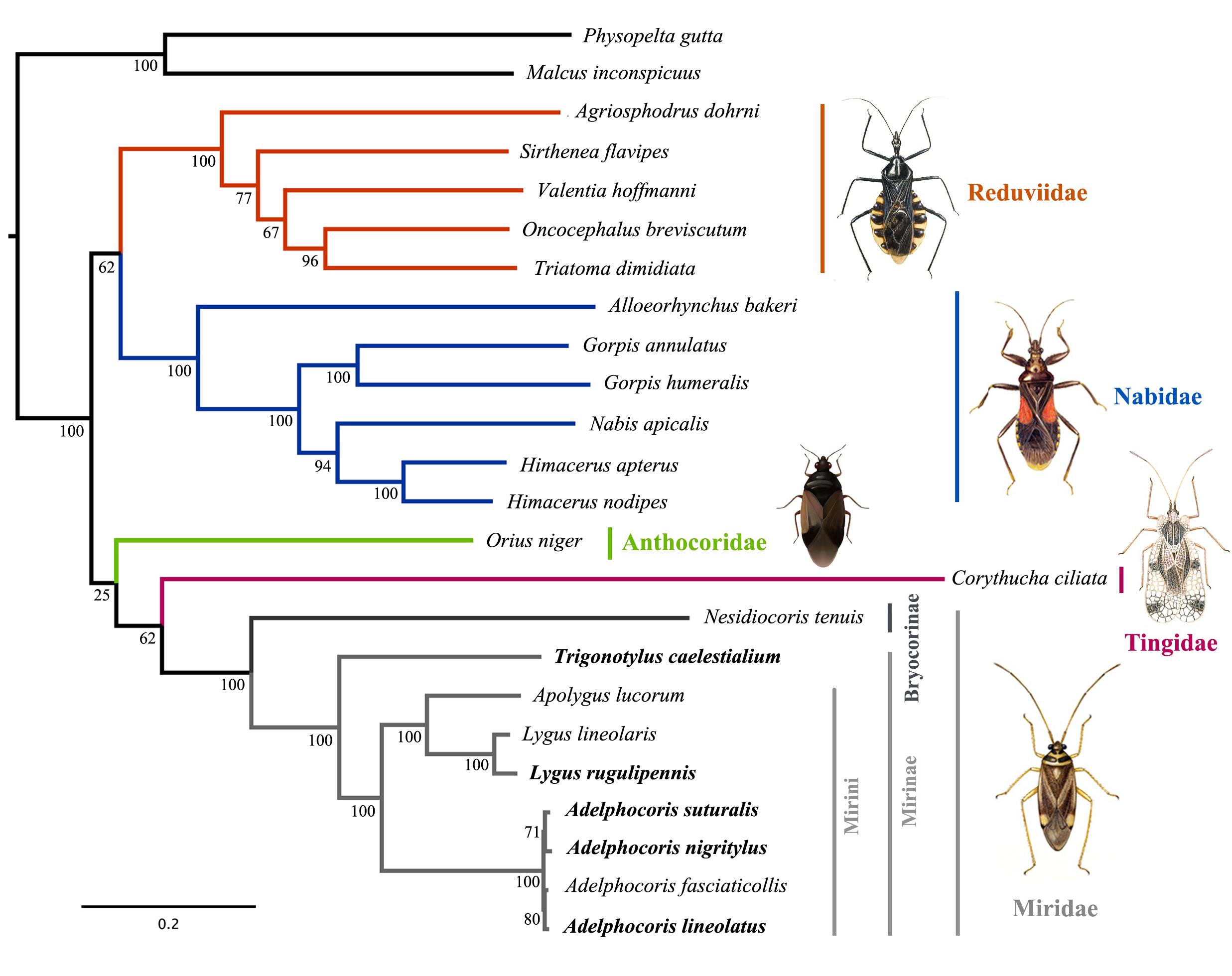

Supplement: Figure S9 — Phylogeny of Cimicomorpha produced from AA (ML). Numbers close to the branching points were ML bootstrap support values. The newly sequenced species were highlighted in bold. (TIF) [file pone.0101375.s009.tif]
