# Supplementary material for: Comparative Mitogenomics of Plant Bugs (Hemiptera: Miridae): Identifying the AGG Codon Reassignments between Serine and Lysine
Source: PLoS One. 2014 Jul 2;9(7):e101375. doi: 10.1371/journal.pone.0101375 (PMC4079613; doi:10.1371/journal.pone.0101375)
Supplement: Table S1 — Collection information of plant bugs sequenced in this study. (DOCX) [file pone.0101375.s010.docx]

**Table S1 Collection information of plant bugs newly sequenced in the present study**

| **Species** | **Locality** | **Time** |
| --- | --- | --- |
| *Adelphocoris lineolatus* | Yuncheng, Shanxi (35°11' N, 111°05' E) | 7, July 2011 |
| *Adelphocoris nigritylus* | Hengshui, Hebei (37°42' N, 115°48' E) | 24, July 2011 |
| *Adelphocoris suturalis* | Xiangyang, Hubei (32°12' N, 112°23' E) | 16, August 2011 |
| *Lygus rugulipennis* | Alaer, Xinjiang (40°33' N, 81°18' E) | 6, August 2010 |
| *Trigonotylus caelestialium* | Haidian, Beijing (40°02' N, 116°17' E) | 22, June 2011 |
