# Supplementary material for: Comparative Mitogenomics of Plant Bugs (Hemiptera: Miridae): Identifying the AGG Codon Reassignments between Serine and Lysine
Source: PLoS One. 2014 Jul 2;9(7):e101375. doi: 10.1371/journal.pone.0101375 (PMC4079613; doi:10.1371/journal.pone.0101375)
Supplement: Table S2 — Primers used in this study. (DOCX) [file pone.0101375.s011.docx]

**Table S2 Primers used in this study**

|  |  | **Primers used for *Adelphocoris suturalis*** |  |
| --- | --- | --- | --- |
| **Fragment no.** | **Primer name** | **Nucleotide sequence (5’-3’)** | **Reference** |
| 1 | TI-J34  TW-N1284 | GCCTGAAAAAGGGTTACTTTGATA  ACARCTTTGAAGGYTAWTAGTTT | [24]  [24] |
| 2 | F1-J1284  R1-N1709 | ATTGAGAATAGGAGGACTG  GCTAAATGGAGTGAGAAG | present study  present study |
| 3 | C1-J1709  C1-N2776 | AATTGGTGGTTTTGGAAATTG  GGTAATCAGAGTATCGACG | [24]  [24] |
| 4 | F1-J2675  R1-N2756 | CACGAGCATACTTTACAT  GCTGGTGGTGAGTTT | present study  present study |
| 5 | C1-J2756  C2-N3389 | ACATTTTTTCCTCAACATTT  TATTCATATCTTCAATATCATTGATG | [24]  [24] |
| 6 | F1-J3389  R1-N3790 | GATACCTGCTTGAAGG  TTCTTGTTGATGGGTC | present study present study |
| 7 | TK-J3790  A6-N4552 | CATTAAGTGACTGAAAGCAAGTA  ATGACCTGCAATTATATTAGC | [24]  [24] |
| 8 | F1-J4552  R1-N4792 | GAATAATACCCAGACG  GGGATAATCTACTATGG | present study  present study |
| 9 | C3-J4792  N3-N5731 | GTTGATTATAGACCWTGRCC  TTAGGGTCAAATCCRCAYTC | [24]  [24] |
| 10 | F1-J5731  R1-N7077 | CTGACACTTTGTTGATG  GGATTGGGTGCTAAT | present study  present study |
| 11 | N5-J7077  N5-N7793 | TTAAATCCTTTGAGTAAAATCC  TTAGGTTGAGATGGTTTAGG | [24]  [24] |
| 12 | N5-J7572  N4-N8727 | AAACGGAAACTGAGCTCTCTTAGT  AAATCTTTAATTGCCTATTCTTC | [24]  [24] |
| 13 | F1-J8430  R1-N8601 | CACCTGAAGCACAAT  TTACCAAAGGCTCAT | present study  present study |
| 14 | N4-J8641  N4L-N9629 | CCAGAAGAACATAANCCRTG  GTTTGTGAGGGWGYTTTRGG | [24]  [24] |
| 15 | F1-J9038  R1-N10621 | ATGAGCCTTTGGTAATC  CCCTAAGAATGCTGTT | present study  present study |
| 16 | CB-J10621  CB-N11526 | CTCATACTGATGAAATTTTGGTTC  TTCTACTGGTCGTGCTCCAATTCA | [24]  [24] |
| 17 | CB-J11335  N1-N12067 | CATATTCAACCAGAATGATA  AATCGTTCTCCATTTGATTTTGC | [24]  [24] |
| 18 | N1-J11876  N1-N12595 | CGAGGTAAAGTACCACGTACTCA  GTTGGATTTCTAACTTTATTRGARCG | [24]  [24] |
| 19 | N1-J12261  LR-N13000 | TACCTCATAAGAAATAGTTTGAGC  TTACCTTAGGGATAACAGCGTAA | [24]  [24] |
| 20 | LR-J12888  LR-N13889 | CCGGTCTGAACTCAGATCATGTA  ATTTATTGTACCTTTTGTATCAG | [24]  [24] |
| 21 | SR-J13342  SR-N14220 | CCTTTGCACAGTCAAAATACTGC  TTATGCACACATCGCCCGTC | [24]  [24] |
| 22 | F1-J14220  R1-N14197 | TCTCAAACAACCAGAT  TTCAGAGGAACCTGT | present study  present study |
| 23 | SR-J14197  SR-N14745 | GTAAAYCTACTTTGTTACGACTT  GTGCCAGCAAYCGCGGTTATAC | [24]  [24] |
| 24 | F1-J1  R1-N34 | CTATGGGCAGATAGCTGAACTT  TATGAAATGGTGGTATGCCTAT | present study  present study |
|  |  | **Primers used for *Adelphocoris lineolatus*** |  |
| **Fragment No.** | **Primer name** | **Nucleotide sequence (5’-3’)** | **Reference** |
| 1 | TM-J210  TW-N1284 | AATTAAGCTACTAGGTTCATACCC  ACARCTTTGAAGGYTAWTAGTTT | [24]  [24] |
| 2 | F3-J1284  R3-N1709 | TATTGAGAATAGGAGGAT  TTTGCTGATAATGGTG | present study  present study |
| 3 | C1-J1709  C1-N2776 | AATTGGTGGTTTTGGAAATTG  GGTAATCAGAGTATCGACG | [24]  [24] |
| 4 | F3-J2675  R3-N2756 | TATGAGCAATAGGGTTTG  TTCTTGATCCGATAGATG | present study  present study |
| 5 | C1-J2756  C2-N3389 | ACATTTTTTCCTCAACATTT  TATTCATATCTTCAATATCATTGATG | [24]  [24] |
| 6 | F3-J3389  R3-N3790 | AATGCCAACTTGATCA  ATATGGGCGATATTTG | present study  present study |
| 7 | TK-J3790  N6-N4552 | CATTAAGTGACTGAAAGCAAGTA  ATGACCTGCAATTATATTAGC | [24]  [24] |
| 8 | F3-J4552  R3-J4792 | AACTTTAGCCCTACC  GGGATAATCTACTATGG | present study  present study |
| 9 | C3-J4792  N3-N5731 | GTTGATTATAGACCWTGRCC  TTAGGGTCAAATCCRCAYTC | [24]  [24] |
| 10 | F3-J5731  R3-N7572 | TTGGATTTGAAGCAGCAG  GGGATGGTTTAGGTTTGG | present study  present study |
| 11 | N5-J7572  N4-N8727 | AAACGGAAACTGAGCTCTCTTAGT  AAATCTTTAATTGCCTATTCTTC | [24]  [24] |
| 12 | F3-J8432  R3-N8641 | CACCTGAAGCACAAT  TGATTACCAAAGGCT | present study  present study |
| 13 | N4-8641  CB-10608 | CCAGAAGAACATAANCCRTG  CCAAGTARTGAWCCAAARTTTCA | [24]  [24] |
| 14 | F3-J10608  R3-N11159 | AGAGTAGCATCAAATG GTAAGGTTCTTCTGC | present study  present study |
| 15 | CB-J11335  N1-N12067 | CATATTCAACCAGAATGATA  AATCGTTCTCCATTTGATTTTGC | [24]  [24] |
| 16 | N1-J11876  N1-N12595 | CGAGGTAAAGTACCACGTACTCA  GTTGGATTTCTAACTTTATTRGARCG | [24]  [24] |
| 17 | F3-J12595  R3-N12888 | CATAAACTCCCATTCCAG  AGGACGAGAAGACCCTAT | present study  present study |
| 18 | LR-J12888  LR-N13889 | CCGGTCTGAACTCAGATCATGTA  ATTTATTGTACCTTTTGTATCAG | [24]  [24] |
| 19 | SR-J13342  SR-N14220 | CCTTTGCACAGTCAAAATACTGC  TTATGCACACATCGCCCGTC | [24]  [24] |
| 20 | SR-J14197  SR-N14745 | GTAAAYCTACTTTGTTACGACTT  GTGCCAGCAAYCGCGGTTATAC | [24]  [24] |
| 21 | FC3-J14745  TM-N200 | ATCTACTTTGTTACGACTTATC  ACCTTTATAAATGGGGTATGAACC | present study  [24] |
|  |  | **Primers used for *Adelphocoris nigritylus*** |  |
| **Fragment No.** | **Primer name** | **Nucleotide sequence (5’-3’)** | **Reference** |
| 1 | TM-J210  TW-N1284 | AATTAAGCTACTAGGTTCATACCC  ACARCTTTGAAGGYTAWTAGTTT | [24]  [24] |
| 2 | F4-J1284  R4-N2756 | CTCCCTTTATAGGATTCATACC GAGATTCTTTGACCCGATAG | present study  present study |
| 3 | C1-J2756  C2-N3389 | ACATTTTTTCCTCAACATTT  TATTCATATCTTCAATATCATTGATG | [24]  [24] |
| 4 | F4-J3389  R4-N3790 | GATACCTGCTTGAAGG  TTCTTGTTGATGGGTC | present study  present study |
| 5 | TK-J3790  A6-N4552 | CATTAAGTGACTGAAAGCAAGTA  ATGACCTGCAATTATATTAGC | [24]  [24] |
| 6 | F4-J4552  R4-N4792 | AACTTTAGCCCTACC  GTATGCTTTCCTTGA | present study  present study |
| 7 | C3-J4792  N3-N5731 | GTTGATTATAGACCWTGRCC  TTAGGGTCAAATCCRCAYTC | [24]  [24] |
| 8 | F4-J5731  R4-N7077 | TTGGGTTTGAAGCAGCAG  TTTGGTTACGGCAGGAGT | present study  present study |
| 9 | N5-J7077  N5-N7793 | TTAAATCCTTTGAGTAAAATCC  TTAGGTTGAGATGGTTTAGG | [24]  [24] |
| 10 | N5-J7572  N4-N8727 | AAACGGAAACTGAGCTCTCTTAGT  AAATCTTTAATTGCCTATTCTTC | [24]  [24] |
| 11 | F4-J8430  R4-N8601 | ACCACCTAAAGCACAAT GATTACCAAAGGCTCAT | present study  present study |
| 12 | N6-J8641  N6-N10608 | CCAGAAGAACATAANCCRTG  CCAAGTARTGAWCCAAARTTTCA | [24]  [24] |
| 13 | F4-J10608  R4-N11335 | ATGAGCCTTTGGTAA  GTCCTGTGATAATGTAAG | present study  present study |
| 14 | CB-J11335  N1-N12067 | CATATTCAACCAGAATGATA  AATCGTTCTCCATTTGATTTTGC | [24]  [24] |
| 15 | N1-J11876  N1-N12595 | CGAGGTAAAGTACCACGTACTCA  GTTGGATTTCTAACTTTATTRGARCG | [24]  [24] |
| 16 | N1-J12261  LR-N13000 | TACCTCATAAGAAATAGTTTGAGC  TTACCTTAGGGATAACAGCGTAA | [24]  [24] |
| 17 | LR-J12888  LR-N13889 | CCGGTCTGAACTCAGATCATGTA  ATTTATTGTACCTTTTGTATCAG | [24]  [24] |
| 18 | SR-J13342  SR-N14220 | CCTTTGCACAGTCAAAATACTGC  TTATGCACACATCGCCCGTC | [24]  [24] |
| 19 | F4-J14220  R4-N14197 | TCTCAAACAACCAGAT  GTTAGGTCAAGGTTTAG | present study  present study |
| 20 | SR-J14197  SR-N14745 | GTAAAYCTACTTTGTTACGACTT  GTGCCAGCAAYCGCGGTTATAC | [24]  [24] |
|  |  | **Primers used for** ***Lygus rugulipennis*** |  |
| **Fragment No.** | **Primer name** | **Nucleotide sequence (5’-3’)** | **Reference** |
| 1 | F5-J1  R5-N210 | GATCATATTGCTATGGGCAGAT  TCTGGGAATCATATGTGGAATG | present study  present study |
| 2 | TM-J210  TW-N1284 | AATTAAGCTACTAGGTTCATACCC  ACARCTTTGAAGGYTAWTAGTTT | [24]  [24] |
| 3 | F5-J1284  R5-N2756 | GGATTACCTCCCTTTAT  TCAGCGTATGAATGTTC | present study  present study |
| 4 | C1-J2756  C2-N3665 | ACATTTTTTCCTCAACATTT  CCACAAATTTCTGAACACTG | [24]  [24] |
| 5 | F5-J3665  R5-N4792 | AATACCAGCAATCACCCTAT  CTTGGAATGTCCCTTCTC | present study  present study |
| 6 | C3-J4792  N3-N5731 | GTTGATTATAGACCWTGRCC  TTAGGGTCAAATCCRCAYTC | [24]  [24] |
| 7 | F5-J5731  R5-N6172 | GTTTCTTTATGGCTACTGG  GGATGGAATTATCCCTTA | present study  present study |
| 8 | TN-J6172  N5-N7211 | AGAGGCAATTTATTGTTAATAA  TTAAGGCTTTATTATTTATATGTGC | [24]  [24] |
| 9 | N5-J7077  N5-N7793 | TTAAATCCTTTGAGTAAAATCC  TTAGGTTGAGATGGTTTAGG | [24]  [24] |
| 10 | F5-J7793  R5-N8641 | CCGCAGTTACTAAAGTTGAAG  TGTTGAGGCACCTATTTCT | present study  present study |
| 11 | N4-J8641  N4L-N9629 | CCAGAAGAACATAANCCRTG  GTTTGTGAGGGWGYTTTRGG | [24]  [24] |
| 12 | F5-J9620  R5-N11335 | ATGCCATCAACATTG  TTTATCACTATTGCCC | present study  present study |
| 13 | CB-J11335  N1-N12067 | CATATTCAACCAGAATGATA  AATCGTTCTCCATTTGATTTTGC | [24]  [24] |
| 14 | F5-J12067  R5-N12888 | TACCACGAACCCAAAT  ATAGGACGAGAAGACCCT | present study  present study |
| 15 | LR-J12888  LR-N13889 | CCGGTCTGAACTCAGATCATGTA  ATTTATTGTACCTTTTGTATCAG | [24]  [24] |
| 16 | F5-J13889  R5-N14197 | AATCATAGGGCAGGTTAG  GTAGAGATGGGTTACATTGT | present study  present study |
| 17 | SR-J14197  SR-N14745 | GTAAAYCTACTTTGTTACGACTT  GTGCCAGCAAYCGCGGTTATAC | [24]  [24] |
| 18 | F-14438  R-14694 | GGATTATCAATTAAAGGACAGG TAAGACTTGCGGATATTTATTC | present study  present study |
| 19 | F- C14694  R-C210 | ATCCACAATTGTATAACCGC ATCAAGATAACCCTTTTTGTC | present study  present study |
|  |  | **Primers used for *Trigonotylus caelestialium*** |  |
| **Fragment No.** | **Primer name** | **Nucleotide sequence (5’-3’)** | **Reference** |
| 1 | TI-J34  TW-N1284 | GCCTGAAAAAGGGTTACTTTGATA  ACARCTTTGAAGGYTAWTAGTTT | [24]  [24] |
| 2 | F6-J1284  R6-N2756 | TGCTTTATCAACCCTATCAG  GCACTATTCTGCCACATT | present study  present study |
| 3 | C1-J2756  C2-N3665 | ACATTTTTTCCTCAACATTT  CCACAAATTTCTGAACACTG | [24]  [24] |
| 4 | F6-J3665  R6-N3790 | CTCTACTATTTATCGCTTTACC  TATTCTTGTTGCTGGATCAAAG | present study  present study |
| 5 | TK-J3790  A6-N4552 | CATTAAGTGACTGAAAGCAAGTA  ATGACCTGCAATTATATTAGC | [24]  [24] |
| 6 | F6-J4552  R6-N4792 | GATCCAGCAACAAGAAT  TGTGGAAGAATGCTC | present study  present study |
| 7 | C3-J4792  N3-N5731 | GTTGATTATAGACCWTGRCC  TTAGGGTCAAATCCRCAYTC | [24]  [24] |
| 8 | F6-J5731  R6-N6172 | GAGACTCAGTGTATGGG  AATGGATTCTGGTTG | present study  present study |
| 9 | TN-J6172  N5-N7211 | AGAGGCAATTTATTGTTAATAA  TTAAGGCTTTATTATTTATATGTGC | [24]  [24] |
| 10 | N5-J7077  N5-N7793 | TTAAATCCTTTGAGTAAAATCC  TTAGGTTGAGATGGTTTAGG | [24]  [24] |
| 11 | F6-J7793  R6-N11335 | CATTGCTGCTGGTAATCAAG  TATGGCTCTTCTGCTGGG | present study  present study |
| 12 | CB-J11335  N61N12067 | CATATTCAACCAGAATGATA  AATCGTTCTCCATTTGATTTTGC | [24]  [24] |
| 13 | F6-J12067  R6-N12261 | CCTCGAACCCAAATAAACATAA  AGAGCGTAAGGTGTTAGGTTAT | present study  present study |
| 14 | N1-J12261  LR-N13000 | TACCTCATAAGAAATAGTTTGAGC  TTACCTTAGGGATAACAGCGTAA | [24]  [24] |
| 15 | F6-J13000  R6-N14197 | ACGCTGTTATCCCTAAGG  GTGGAGATGGGTCACATT | present study  present study |
| 16 | SR-J14197  SR-N14745 | GTAAAYCTACTTTGTTACGACTT  GTGCCAGCAAYCGCGGTTATAC | [24]  [24] |
| 17 | FC6-J14745  RC6-N210 | TATTGCTAAACCTTGACC  GGATGCTATTCTTTGTGT | present study  present study |
