# Supplementary material for: Comparative Mitogenomics of Plant Bugs (Hemiptera: Miridae): Identifying the AGG Codon Reassignments between Serine and Lysine
Source: PLoS One. 2014 Jul 2;9(7):e101375. doi: 10.1371/journal.pone.0101375 (PMC4079613; doi:10.1371/journal.pone.0101375)
Supplement: Table S3 — The best partitioning scheme selected by PartitionFinder for different datasets. (DOCX) [file pone.0101375.s012.docx]

**Table S3 The best partitioning scheme selected by PartitionFinder for different dataset**

| **Dataset** | **Subset Partitions** | **Best Model** |
| --- | --- | --- |
| nt123RNA:  6 partitions (BI) | P1: (12S, 16S, ATP6_pos1, ATP8_pos1, ND2_pos1, ND3_pos1, ND6_pos1, Leu_uur) | GTR+I+G |
|  | P2: (ATP6_pos2, COIII_pos2, COII_pos2, COI_pos2, CYTB_pos2, ND1_pos2, ND2_pos2, ND3_pos2, ND4L_pos2, ND4_pos2, ND5_pos2, ND6_pos2) | GTR+I+G |
|  | P3: (ATP6_pos3, ATP8_pos3, COIII_pos3, COII_pos3, COI_pos3, CYTB_pos3, ND2_pos3, ND3_pos3, ND6_pos3) | HKY+I+G |
|  | P4: (ATP8_pos2, COIII_pos1, COII_pos1, COI_pos1, CYTB_pos1, Ala, Arg, Asn, Asp, Cys, Glu, Gly, His, Leu_cun, Lys, Phe, Pro, Ser_agn, Ser_ucn, Thr, Trp, Tyr, Val) | GTR+I+G |
|  | P5: (ND1_pos1, ND4L_pos1, ND4_pos1, ND5_pos1) | GTR+I+G |
|  | P6: (ND1_pos3, ND4L_pos3, ND4_pos3, ND5_pos3) | HKY+I+G |
| nt123RNA:  6 partitions (ML) | P1: (12S, 16S, ATP8_pos1, ND2_pos1, ND3_pos1, ND6_pos1) | GTR+I+G |
|  | P2: (ATP6_pos1, ATP8_pos2, COIII_pos1, COII_pos1, COI_pos1, CYTB_pos1, Ala, Arg, Asn, Asp, Cys, Glu, Gly, His, Leu_cun, Leu_uur, Lys, Phe, Pro, Ser_agn, Ser_ucn, Thr, Trp, Tyr, Val) | GTR+I+G |
|  | P3: (ATP6_pos2, COIII_pos2, COII_pos2, COI_pos2, CYTB_pos2, ND1_pos2, ND2_pos2, ND3_pos2, ND4L_pos2, ND4_pos2, ND5_pos2, ND6_pos2) | GTR+I+G |
|  | P4: (ATP6_pos3, ATP8_pos3, COIII_pos3, COII_pos3, COI_pos3, CYTB_pos3, ND2_pos3, ND3_pos3, ND6_pos3) | GTR+I+G |
|  | P5: (ND1_pos1, ND4L_pos1, ND4_pos1, ND5_pos1) | GTR+I+G |
|  | P6: (ND1_pos3, ND4L_pos3, ND4_pos3, ND5_pos3) | GTR+I+G |
| nt123:  5 partitions (BI) | P1: (ATP6_pos1, ATP8_pos1, ND1_pos1, ND2_pos1, ND3_pos1, ND4L_pos1, ND4_pos1, ND5_pos1, ND6_pos1) | GTR+I+G |
|  | P2: (ATP6_pos2, ATP8_pos2, COIII_pos2, COII_pos2, COI_pos2, CYTB_pos2, ND1_pos2, ND2_pos2, ND3_pos2, ND4L_pos2, ND4_pos2, ND5_pos2, ND6_pos2) | GTR+I+G |
|  | P3: (ATP6_pos3, ATP8_pos3, COIII_pos3, COII_pos3, COI_pos3, CYTB_pos3, ND2_pos3, ND3_pos3, ND6_pos3) | HKY+I+G |
|  | P4: (COIII_pos1, COII_pos1, COI_pos1, CYTB_pos1) | GTR+I+G |
|  | P5: (ND1_pos3, ND4L_pos3, ND4_pos3, ND5_pos3) | HKY+I+G |
| nt123:  5 partitions (ML) | P1: (ATP6_pos1, COIII_pos1, COII_pos1, COI_pos1, CYTB_pos1) | GTR+I+G |
|  | P2: (ATP6_pos2, ATP8_pos2, COIII_pos2, COII_pos2, COI_pos2, CYTB_pos2, ND1_pos2, ND2_pos2, ND3_pos2, ND4L_pos2, ND4_pos2, ND5_pos2, ND6_pos2) | GTR+I+G |
|  | P3: (ATP6_pos3, ATP8_pos3, COIII_pos3, COII_pos3, COI_pos3, CYTB_pos3, ND2_pos3, ND3_pos3, ND6_pos3) | GTR+I+G |
|  | P4: (ATP8_pos1, ND1_pos1, ND2_pos1, ND3_pos1, ND4L_pos1, ND4_pos1, ND5_pos1, ND6_pos1) | GTR+I+G |
|  | P5: (ND1_pos3, ND4L_pos3, ND4_pos3, ND5_pos3) | GTR+I+G |
| RNA: 2 partitions | P1: (12S, 16S) | GTR+I+G |
|  | P2: (Ala, Arg, Asn, Asp, Cys, Glu, Gly, His, Leu_cun, Leu_uur, Lys, Phe, Pro, Ser_agn, Ser_ucn, Thr, Trp, Tyr, Val) | GTR+I+G |
| AA: 2 partitions | P1: (ATP6, ATP8, COIII, COII, COI, CYTB, ND2, ND3, ND6) | mtREV+I+G (BI)  MtArt+I+G (ML) |
|  | P2: (ND1, ND4L, ND4, ND5) | mtREV+I+G (BI)  MtArt+I+G (ML) |
