# Supplementary material for: Comparative Mitogenomics of Plant Bugs (Hemiptera: Miridae): Identifying the AGG Codon Reassignments between Serine and Lysine
Source: PLoS One. 2014 Jul 2;9(7):e101375. doi: 10.1371/journal.pone.0101375 (PMC4079613; doi:10.1371/journal.pone.0101375)
Supplement: Table S4 — Structural features of plant bug mitochondrial genomes. (DOC) [file pone.0101375.s013.doc]

**Table S4 Structural features of plant bug mitochondrial genomes**

|  |  |  | **A+T (%)** | | ***lrRNA*** | | ***srRNA*** | | **Control region** | |
| --- | --- | --- | --- | --- | --- | --- | --- | --- | --- | --- |
|  | **Size (bp)** | **Gene order ^a^** | **Genome** | **PCGs** | **Size (bp)** | **A+T%** | **Size (bp)** | **A+T%** | **Size (bp)** | **A+T%** |
| *Apolygus lucorum* | 14768 | A | 76.8 | 75.8 | 1247 | 80.9 | 794 | 79.1 | 228 | 81.1 |
| *Lygus lineolaris* | 17027 | A | 76.1 | 74.8 | 1248 | 79.9 | 844 | 78.9 | 2318 | 77.2 |
| *Lygus rugulipennis* | >15819 | A | N/A | 74.5 | 1250 | 79.7 | 824 | 78.2 | >1125 | N/A |
| *Nesidiocoris tenuis* | 17544 | A | 75.0 | 73.3 | 1221 | 78.2 | 798 | 75.4 | 3155 | 78.1 |
| *Adelphocoris fasciaticollis* | 15434 | A | 77.4 | 76.3 | 1229 | 81.1 | 793 | 81.3 | 922 | 78.2 |
| *Adelphocoris lineolatus* | 15595 | A | 77.1 | 76.0 | 1231 | 80.4 | 789 | 81.3 | 1078 | 77.4 |
| *Adelphocoris nigritylus* | >14522 | A | N/A | 76.2 | 1231 | 81.4 | 789 | 81.1 | N/A | N/A |
| *Adelphocoris suturalis* | >14327 | A | N/A | 76.0 | 1230 | 80.8 | >595 | N/A | N/A | N/A |
| *Trigonotylus caelestialium* | >15095 | A | N/A | 74.4 | 1238 | 77.0 | >592 | N/A | >560 | N/A |

^a^ A = ancestral arrangement; N/A a = not available.
