# Supplementary material for: Comparative Mitogenomics of Plant Bugs (Hemiptera: Miridae): Identifying the AGG Codon Reassignments between Serine and Lysine
Source: PLoS One. 2014 Jul 2;9(7):e101375. doi: 10.1371/journal.pone.0101375 (PMC4079613; doi:10.1371/journal.pone.0101375)
Supplement: Table S5 — Statistics on non-coding sequences in plant bug mitochondrial genomes. (DOC) [file pone.0101375.s014.doc]

**Table S5 Statistics on non-coding sequences in plant bug mitochondrial genomes**

|  | **Proportion (%) ^a^** | **Total length (bp)** | **Non-coding region (bp)** | | | | | |
| --- | --- | --- | --- | --- | --- | --- | --- | --- |
|  |  |  | ***tRNA^Lys^-***  ***tRNA^Asp^*** | ***tRNA^Glu^ -***  ***tRNA^Phe^*** | ***tRNA^Pro^ -***  ***ND6*** | ***ND6-***  ***CytB*** | ***tRNA^Ser(AGN)^ -***  ***ND1*** | ***srRNA - tRNA^Ile^***  ***(CR)*** |
| *Ap. lucorum* | 1.59 | 235 | - | - | - | - | 7 | 228 |
| *Ad. fasciaticollis* | 6.10 | 942 | - | 10 | 2 | 1 | 7 | 922 |
| *Ad. lineolatus* | 7.07 | 1103 | - | 15 | 2 | 1 | 7 | 1078 |
| *Ad.nigritylus** | N/A | >31 | - | 21 | 2 | 1 | 7 | ? |
| *Ad. suturalis** | N/A | >30 | - | 20 | 2 | 1 | 7 | ? |
| *L.rugulipennis** | N/A | >1261 | 2 | - | 1 | 126 | 7 | >1125 |
| *L. lineolaris* | 14.30 | 2440 | 2 | - | 1 | 112 | 7 | 2318 |
| *N. tenuis* | 18.03 | 3163 | - | - | 1 | - | 7 | 3155 |
| *T. coelestialium** | N/A | >780 | 46 | - | 167 | - | 7 | >560 |

* nearly complete mt genome; ^a^ the proportion of total non-coding length to the complete genome length; N/A = not available; - = not present; ? = unknown.
